# Strain‐Release Driven Arsenium Ion Bond Insertion

**DOI:** 10.1002/anie.202510186

**Published:** 2025-07-24

**Authors:** Christoph Riesinger, Florian Meurer, Lisa Zimmermann, Luis Dütsch, Manfred Scheer

**Affiliations:** ^1^ Institute of Inorganic Chemistry University of Regensburg Universitätsstr. 31 93053 Regensburg Germany

**Keywords:** Arsenium ion, Bond insertion, Quantum crystallography, Ring strain, Strain‐release

## Abstract

Although it marks a cornerstone of pnictogenium ion [R_2_Pn]^+^ reactivity, the insertion of arsenium ions [R_2_As]^+^ into non‐polar bonds remains highly challenging. Herein, a synthetic approach is developed, which circumvents the limitations of insertion reactivity of [R_2_As]^+^ (e.g., formal redox state of +V at As) via alleviation of ring strain in the substrate. Thus, unlocking arsenium ion bond insertion delivers the ring‐expanded complexes [{L_n_M}(η^3^‐Pn_3_AsCy_2_][TEF] ({L_n_M} = Cp^‴^Ni, Pn = P (**1**); {L_n_M} = {CpMo(CO)_2_}, Pn = P (**2**), As (**5**); Cp^‴^ = 1,2,4‐^*t*^Bu_3_C_5_H_2_, [TEF]^−^ = [Al{OC(CF_3_)_3_}_4_]^−^). Computational analysis of the reaction mechanism and quantum crystallographic investigation of **1** highlight the release of ring strain as the crucial driving force for this reactivity. This rational is corroborated by the isolation of the arsenium ion coordinated [{CpMo(CO)_2_}_2_(μ,η^2:2^‐P_2_AsCy_2_)][TEF] (**3**) as well as the phosphenium ion inserted [{CpMo(CO)_2_}(η^3^‐As_3_PPh_2_)][TEF] (**4**).

## Graphical abstract

The release of ring strain in *cyclo*‐Pn_3_ (Pn = P, As) ligands is exploited to achieve insertion of arsenium ions [R_2_As]^+^ (R = organic substituent) into non‐polar bonds. This concept is utilized to access complexes bearing exotic *cyclo*‐Pn_3_AsR_2_ ligands. Quantum crystallography and computational analysis shed light on the key energetic contributions unlocking this fundamental mode of reactivity.

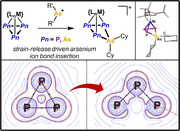

Since their first postulation^[^ ^1^ ^]^ and later isolation,^[^ ^2^, ^3^ ^]^ carbenes and their complexes have evolved into an indispensable class of compounds in organic and organometallic chemistry. With the introduction of stable singlet carbenes,^[^ ^4^, ^5^, ^6^ ^]^ their primary role as ligands was superseded by applications ranging from (organo‐)catalysis,^[^ ^7^, ^8^, ^9^ ^]^ to materials chemistry.^[^ ^10^, ^11^, ^12^ ^]^ Moreover, the past two decades have seen the periodic table gradually being filled in with carbene analogs of other, heavier p‐block elements.^[^ ^13^, ^14^ ^]^ Anionic carbene analogs can be found for example in aluminyl anions, marking one of the most recent additions to this field.^[^ ^15^, ^16^ ^]^ On the other hand, group 15 representatives have seen a recent gain in interest with the isolation of singly substituted pnictinidenes^[^ ^17^, ^18^, ^19^, ^20^ ^]^ and even a triplet nitrene.^[^ ^21^, ^22^ ^]^ Notably, donor free pnictogenium ions (Scheme 1a), the positively charged group 15 carbene analogs, have been isolated only within the past 5 years.^[^ ^23^, ^24^, ^25^ ^]^ This is despite the isolobal principle^[^ ^26^ ^]^ connecting these species to carbenes. Nevertheless, geometrically constrained phosphenium ions ([R_2_P]^+^) have been demonstrated to insert into C─H bonds^[^ ^27^ ^]^ and even catalyze the hydrogenation of unsaturated substrates.^[^ ^28^ ^]^ While previously this reactivity appeared to be reserved to transition metal (TM) catalysts,^[^ ^29^ ^]^ it is enabled by phosphenium ions readily inserting into polar and non‐polar bonds. This propensity to undergo bond insertion has also been utilized widely in organophosphorus chemistry,^[^ ^30^, ^31^ ^]^ and to access unprecedented polyphosphorus cations (Scheme 1b).^[^ ^32^, ^33^, ^34^, ^35^, ^36^, ^37^ ^]^ The reactivity of arsenium ions ([R_2_As]^+^), the heavier analogs of [R_2_P]^+^, is far less explored.^[^ ^38^, ^39^, ^40^, ^41^, ^42^, ^43^, ^44^, ^45^, ^46^ ^]^ This is despite organo‐arsenic compounds holding significant application in drug design^[^ ^47^, ^48^ ^]^ or MOVPE (metal‐organic vapor phase epitaxy) processes for semiconductor manufacturing.^[^ ^49^, ^50^ ^]^ A major drawback of arsenium ions is their articulated restriction to coordination of Lewis bases and the lack of bond insertion reactivity, excluding them from many catalytic applications (*vide supra*, Scheme 1b).^[^ ^35^, ^42^ ^]^ Generally, this deficiency can be attributed to the inferior accessibility of the As(+V) redox state compared to e.g. P(+V). A clear example for this issue is demonstrated by comparing the intramolecular reactivity of transient di‐terphenyl phosphenium and arsenium ions.^[^ ^43^, ^46^ ^]^

**Scheme 1:**
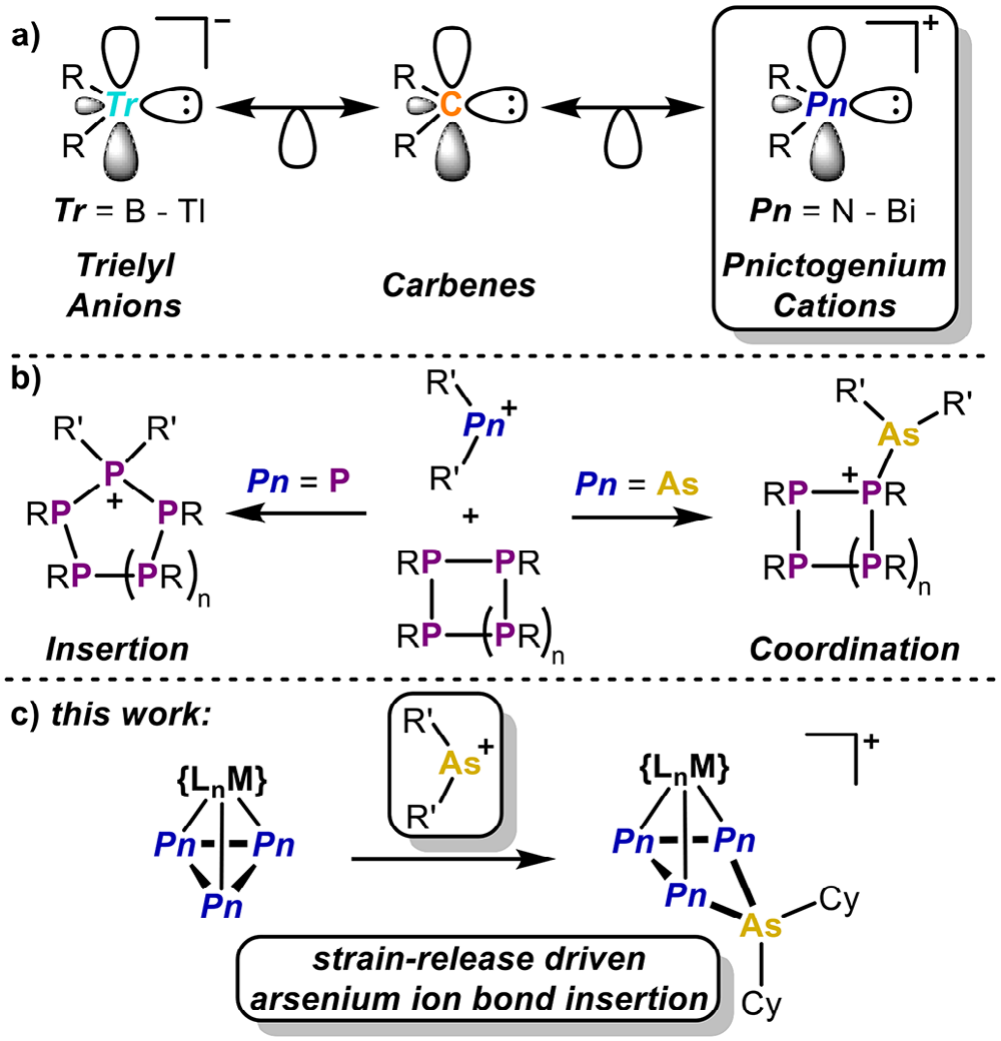
a) Isolobal relationship between carbenes and their ionic group 13 and group 15 analogs; b) reactivity of phosphenium and arsenium ions toward polyphosphorus species (e.g. R = cyclohexyl, *n* = 1, [R′_2_P]^+^ = [Me_2_P]^+^, [R′_2_As]^+^ = [HN(o‐C_6_H_4_)_2_As]^+^); c) release of ring strain in three‐membered polypnictogen ligands drives the insertion of arsenium ions.

A similar trend is observed when pnictogenium ions are reacted with polyphosphorus (P_n_) ligand complexes. While phosphenium ions readily insert into one of the P─P bonds,^[^ ^51^, ^52^, ^53^ ^]^ arsenium ions are found to only coordinate to one of the respective P atoms.^[^ ^54^, ^55^ ^]^ However, when reacting arsenium ions with comparably small *cyclo*‐P_n_ ligands (e.g. *n* = 4), spectroscopic data suggests bond insertion to be in reach at least in an equilibrium, which could however not be structurally validated.^[^ ^52^ ^]^

This led to the hypothesis that the bond insertion reactivity of arsenium ions may ultimately be achievable, by allowing the release of ring strain within a substrate to drive the reaction (Scheme 1c). In case of success, this fundamental mode of reactivity could be the initial step toward unprecedented arsenium ion redox catalysis and beyond that pioneer a new avenue into the preparation of organo‐arsenic compounds. The latter becomes even more apparent when considering the recent surge in popularity of small, strained molecules, such as *bicyclo*‐butanes (BCBs)^[^ ^56^, ^57^ ^]^ or *cyclo*‐propanes, within organic chemistry.

Herein, a synthetic strategy is developed enabling arsenium ion bond insertion through the release of ring strain within the substrate. This methodology grants access to the first structurally authenticated products of arsenium ion bond insertion. Complexes of highly strained *cyclo*‐P_3_ ligands ([{L_n_M}(η^3^‐P_3_)] {L_n_M} = {Cp^‴^Ni} (**A_Ni_**)^[^ ^58^ ^]^ {CpMo(CO)_2_} (**A_Mo_**);^[^ ^59^ ^]^ Cp^‴^ = 1,2,4‐^t^Bu_3_C_5_H_2_, Cp = C_5_H_5_) were selected as model targets based on their established reactivity toward phosphenium ions^[^ ^60^ ^]^ and considering the ambiguous equilibrium reactivity of arsenium ions toward *cyclo*‐P_4_ derivatives (*vide supra*).^[^ ^52^ ^]^

Intriguingly, reacting **A_M_** (M = Ni, Mo) with prototypical [Cy_2_As][TEF], generated in situ from Cy_2_AsBr and Tl[TEF], leads to a color change from orange/yellow to red (**A_Ni_**) or orange (**A_Mo_**), respectively. The ^31^P NMR spectra of the crude reaction mixtures reveal the consumption of the starting materials (see ESI), which is accompanied by the emergence of a doublet and a triplet shifted to higher fields, indicating the insertion of the arsenium ion [Cy_2_As]^+^ into the *cyclo*‐P_3_ ligand. The resulting *cyclo*‐P_3_AsCy_2_ complexes [{Cp^‴^Ni}(η^3^‐P_3_AsCy_2_)][TEF] (**1**, Figure 1a) and [{CpMo(CO)_2_}(η^3^‐P_3_AsCy_2_)][TEF] (**2**) could be isolated as red or orange solids in good yields of 75% (**1**) and 85% (**2**) after workup, respectively. Both species are highly sensitive toward air, moisture, and elevated temperatures. The latter necessitates workup and storage of **1** and **2** at a maximum of 0 °C. Otherwise, these species decompose both in solution as well as the solid state. In case of **1**, this decomposition could be traced to afford the known triple decker ion [{Cp^‴^Ni}_2_(μ,η^3:3^‐P_3_)]^+[^ ^51^ ^]^ in addition to a mixture of intractable side‐products (see ESI). Notably, the insertion of arsenium ions into P_4_ (which is isolobal to **A_M_**) could not be achieved previously,^[^ ^61^ ^]^ which may be attributed to the spherical aromaticity of this molecule counteracting its ring strain.^[^ ^62^ ^]^ Similarly, a mixture of Cy_2_AsBr, Tl[TEF] and P_4_ did not afford arsenium ion insertion, even at elevated temperatures.

**Figure 1:**
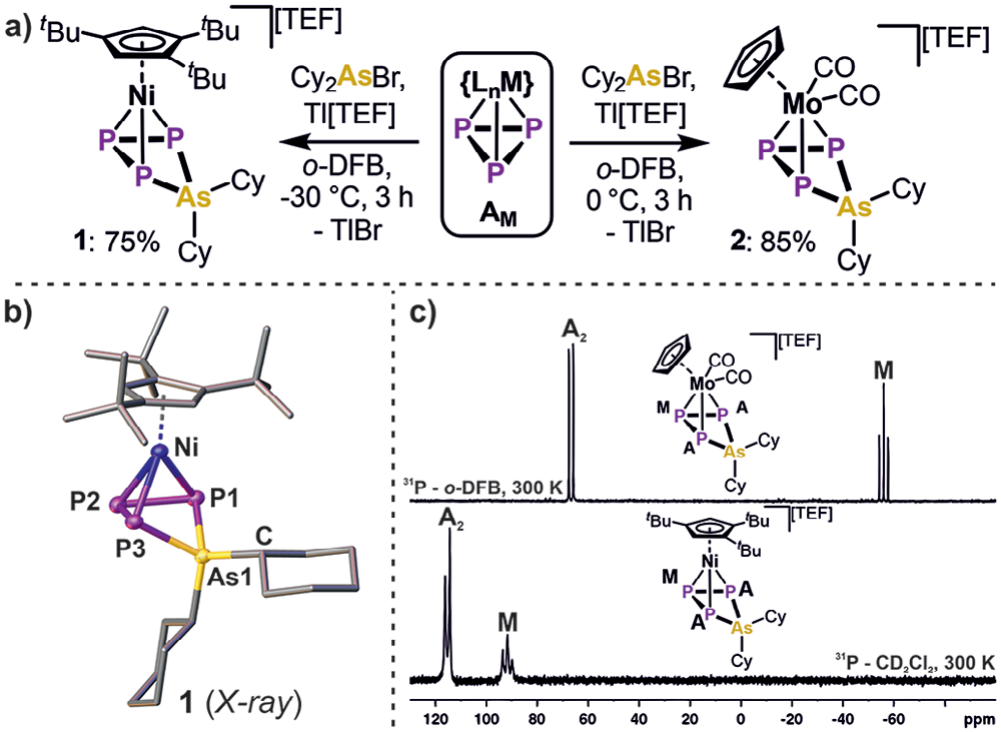
a) Synthesis of *cyclo*‐P_3_AsCy_2_ ligand complexes **1** and **2** via arsenium ion insertion into the *cyclo*‐P_3_ complexes [{Cp^‴^Ni}(η^3^‐P_3_)] (**A_Ni_**, Cp^‴^ = 1,2,4‐^t^Bu_3_C_5_H_2_) and [{CpMo(CO)_2_}(η^3^‐P_3_)] (**A_Mo_**, Cp = C_5_H_5_); b) molecular structure of **1** in the solid state with hydrogen atoms and the counter anion being omitted for clarity, anisotropic displacement parameters are drawn at the 50% probability level; c) ^31^P NMR spectra of **1** and **2** recorded at 300 K with the corresponding assignment of signals.

To structurally confirm the insertion of the [Cy_2_As]^+^ arsenium ion into the *cyclo*‐P_3_ ligand, single crystals of **1** were grown at −30 °C. Indeed, the solid‐state structure of **1** (Figure 1b) reveals a bent *cyclo*‐P_3_AsCy_2_ ligand coordinated to the {Cp^‴^Ni} moiety. Thus, it demonstrates the first structural proof of an arsenium ion bond insertion. The P─P bond lengths in **1** are virtually equivalent (2.190(1) Å) and correspond to slightly shortened P─P single bonds (2.22 Å),^[^ ^63^ ^]^ which compares well to the recently reported *cyclo*‐P_4_R_2_ analogs.^[^ ^51^ ^]^ Similarly, the As1─P1/3 bond lengths (2.296(1)/2.303(1) Å) are in the range of single bonds (2.32 Å),^[^ ^63^ ^]^ completing the four‐membered P_3_As‐cycle. Notably, the P1–P3 distance (3.064(1) Å) clearly indicates bond cleavage and thus confirms the insertion of the arsenium ion into this bond. The ^31^P NMR spectra of **1** and **2** (recorded at 300 K immediately after dissolution) both reveal an A_2_M spin system featuring a doublet and a triplet (Figure 1c), centered at *δ*/ ppm = 115.2, 91.6 (**1**) and 67.7, ‐58.0 (**2**) with coupling constants of ^1^ *J*_P_‐_P_ = 298 Hz (**1**) and 280 Hz (**2**), respectively. While the signals for **1** are broadened due to the partially hindered rotation of the Cp^‴^ ligand, the sharp signals of **2** are in the same chemical shift region as the corresponding ones of their *cyclo*‐P_4_R_2_ analogs.^[^ ^51^, ^60^ ^]^ Although single crystals of **2** could not be obtained, this spectroscopic data confirms the insertion of the arsenium ion into **A_Mo_** as well. This is further substantiated by comparison to the ^31^P NMR spectrum of its Cp* congener (Cp* = C_5_Me_5_), where insertion is prevented based on steric reasons, revealing only a highly broadened signal at *δ*/ ppm = −305 (see Figure S10). In addition, [{CpMo(CO)_2_}(μ,η^2:2^‐P_2_)]^[^ ^59^ ^]^ was reacted with in situ generated [Cy_2_As]^+^. The replacement of one P atom with a {CpMo(CO)_2_} unit in this substrate leads to significant decrease of ring strain through a more delocalized bonding situation. Consequently, the arsenium ion only coordinates to one of the P atoms in [{CpMo(CO)_2_}_2_(μ,η^2:2^‐P_2_AsCy_2_)][TEF] (**3**), which could be isolated in 39% crystalline yield. On the one hand, comparison of the spectroscopic data of **3**, showing two significantly broadened signals at *δ*/ ppm  = −79.0 and −122.4, respectively, consolidates the structural assignment for **2**. On the other hand, this proofs the formation of **1** and **2** to be mainly driven by the release of ring strain in the *cyclo*‐P_3_ starting materials **A_M_**.

To gain further insight into this reactivity, the reaction pathway leading to the formation of **1** and **2** was analyzed computationally on a model system (Cp^‴^ was replaced by Cp and the Cy groups were changed for Me, *ω*B97X‐D4/def2‐TZVP, PCM CH_2_Cl_2_, Figure 2). Initial arsenium ion [Me_2_As]^+^ coordination to **A′_M_** occurs barrierless and formation of the adducts **1′_INT_** and **2′_INT_** is highly exergonic by 95.6 kJ mol^−1^ and 130.9 kJ mol^−1^, respectively. Notably, such preliminary coordination is unfavorable for P_4_ (s‐character of the lone pairs) providing another potential explanation for it not showing the desired reactivity. However, after coordination to **A_M_**, the energetic barriers of 35.6 kJ mol^−1^ (**1′_TS_**) and 54.7 kJ mol^−1^ (**2′_TS_**) for the arsenium ion to undergo subsequent P─P bond insertion are comparably low. Notably, these TS are much more reminiscent of the product (late TS) compared to phosphenium ion insertion into **A′_Ni_**,^[^ ^51^ ^]^ which is exemplified in the increased P1–P3 distance (2.453 Å (**1′_TS_**), 2.576 Å (**2′_TS_**), 2.329 Å (**A′_Ni_** + PMe_2_^+^),^[^ ^51^ ^]^ see ESI for details). Thus, releasing the ring strain of the *cyclo*‐P_3_ ligand in **1′_TS_**/**2′_TS_** appears to compensate the energetic disadvantage of breaking a P─P bond in favor of forming a P─As bond, as well as the emergence of formal arsonium character (As(+V)) on As. Finally, the products **1′** and **2′** are 77.8 kJ mol^−1^ and 54.2 kJ mol^−1^ more exergonic than the coordinated species **1′_INT_**/**2′_INT_**, respectively. This is in stark contrast to all compounds bearing coordinated arsenium ions at a polyphosphorus ligand^[^ ^52^, ^54^, ^55^ ^]^ and highlights this reactivity being driven by the strain‐release from the *cyclo*‐P_3_ ligand. Furthermore, the formation of isomers, in which the arsenium ion is inserted into one of the P─M (M═Mo, Ni) bonds is energetically unfavorable compared to **1′** and **2′** by 2.6 kJ mol^−1^ (**1′_ISO_**, 49.22 kJ mol^−1^ taking Cp^‴^ and Cy‐residues into account, see ESI for details) and 32.1 kJ mol^−1^ (**2′_ISO_**), respectively. Thus, isomerization, as is observed for neutral Co complexes bearing *cyclo*‐P_4_R_2_ ligands, is inconceivable.^[^ ^64^ ^]^

**Figure 2:**
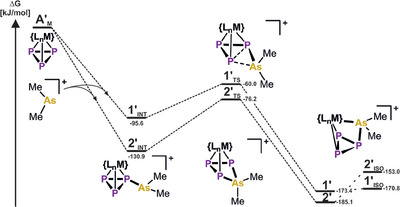
Computed reaction pathway for a model system for the insertion of the [Me_2_As]^+^ arsenium ion into the *cyclo*‐P_3_ ligand complexes **A′_M_** (M = Ni, Mo) to afford the *cyclo*‐P_3_AsMe_2_ complexes **1′** and **2′**; {L_n_M} = {CpNi} (**A′_N_**_i_), {CpMo(CO)_2_} (**A′_Mo_**); computations were performed at the *ω*B97X‐D4/def2‐TZVP (PCM CH_2_Cl_2_) level of theory.

Taking an even closer look at the driving force of this reaction, the release of ring strain can also be visualized experimentally in the topology of the total electron density following a quantum crystallographic Hirshfeld‐Atom‐Refinement (HAR, see Figure S32)^[^ ^65^, ^66^ ^]^ of the structures of **A_Ni_** ^[^ ^67^ ^]^ and **1** (Figure 3a,b). The *cyclo*‐P_3_ bond‐critical points (BCPs) in **A_Ni_** are significantly shifted outside the direct interatomic paths concerning the *cyclo*‐P_3_ triangle compared to the P_3_ plane in **1**. After the arsenium ion insertion, the third P─P covalent BCP vanishes and the P1‐P2‐P3 angle is significantly opened, changing from 60.21(1)° to 88.79(3)°. The deformation density plots (Figure 3c,d) reveal similarities between the P2 atom in **1** and P atoms in general in **A_Ni_**. In contrast, P1 and P3 show tilted lone‐pair density following the ring insertion. There is generally less charge shift from the phosphorus cores to the bonds in **1** compared to **A_Ni_**. Moreover, the As atom in **1** shows a strong charge shift from the core region into its four covalent bonds, which is in line with its formal arsonium (As(+V)) character. Contrastingly, the insertion into the P─P bond significantly lowers the Bader charge^[^ ^68^ ^]^ of As from + 1.13 *e* in [Cy_2_As]^+^ to + 0.82 *e* in **1** (see ESI for details). Nonetheless, the As atom holds the majority of the positive charge (+0.82 *e*) in **1**, while the Ni atomic charge remains almost unchanged (+0.42 *e* in **A_Ni_** and + 0.45 *e* in **1**). Although, this charge state (+0.82 e) is much lower compared to simple organo‐arsonium ions (e.g. Cy_4_As^+^, +1.28 *e*), it corroborates the formal arsonium character in **1**. Conclusively, this analysis discloses the release of ring strain from the *cyclo*‐P_3_ ligand in **A_Ni_** to be the main driving force for arsenium ion bond insertion to afford **1**.

**Figure 3:**
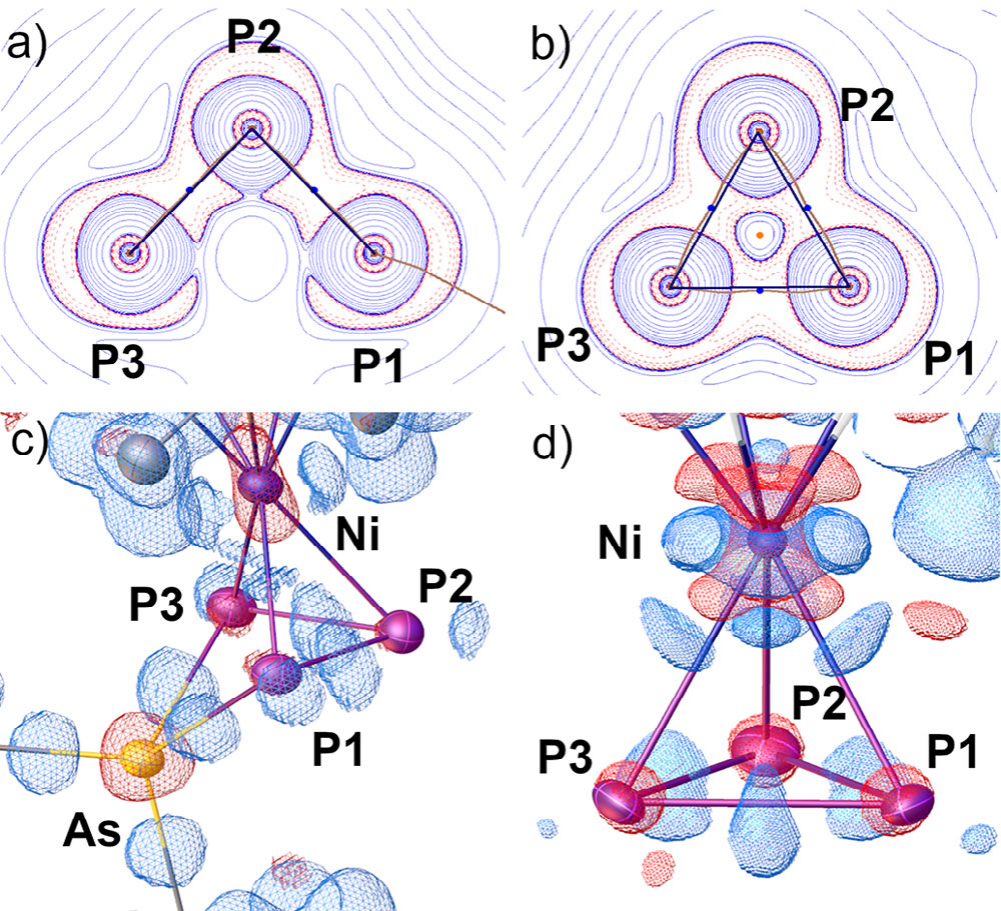
Compared results of the quantum crystallographic comparison of **1** (a and c) and **A_Ni_** (b and d)^[^ ^67^ ^]^: Contour plot of the Laplacian of the total electron density in *e* Å^−5^ and logarithmic iso‐levels in each of the P_3_ planes (a and b) with topological bond paths in orange and interatomic paths in black. Blue dots indicate bond‐critical points, orange dots indicate ring‐critical points. The deformation density is shown at the 0.1 *e* Å^−3^ iso‐level at the NiP_3_As in **1** and the NiP_3_ unit in **A_Ni_**. Blue indicates positive values for the Laplacian plots, indicating valence shell charge depletion, and positive values in the deformation density, indicating more electron density compared to the spherical atomic description, red indicates negative values of the Laplacian plots, indicating valence shell charge accumulation and negative values in the deformation density, indicating less electron density compared to the spherical atomic description.

Finally, the synthetic protocol for strain‐release driven arsenium ion bond insertion developed herein, was sought to be broadened in its scope. To test the transferability of this approach, the *cyclo*‐As_3_ complex [{CpMo(CO)_2_}(η^3^‐As_3_)]^[^ ^69^ ^]^ (**B_Mo_**) appeared to be an optimal candidate. As the cationic functionalization of substituent free polyarsenic ligands is unprecedented so far, **B_Mo_** was initially reacted with the in situ generated [Ph_2_P][TEF]. Interestingly, this afforded [{CpMo(CO)_2_}(η^3^‐As_3_PPh_2_)][TEF] (**4**) in yields of 60% after workup (Figure 4). Notably, **4** is the first representative of a phosphenium ion being inserted into an As─As bond of a polyarsenic ligand and displays a rare example of mixed polypnictogen ligand complexes resulting from electrophilic functionalization.^[^ ^64^ ^]^ **B_Mo_** also reacts with in situ generated [Cy_2_As][TEF] via insertion of the arsenium ion into the *cyclo*‐As_3_ ligand. After workup, the product [{CpMo(CO)_2_}(η^3^‐As_4_Cy_2_)][TEF] (**5**) could be isolated in 69% yield, featuring an unprecedented *cyclo*‐As_4_Cy_2_ ligand. The central MoAs_3_PnR_2_ units of **4** and **5** are isostructural to **1** and their all‐phosphorus analogs.^[^ ^51^, ^60^ ^]^ The As2─As1/3 bonds (2.419(1) Å and 2.431(1) Å) as well as the P1─As1/3 bonds (2.294(2) Å) in **4** can be considered slightly elongated single bonds.^[^ ^63^ ^]^ In agreement with the insertion of the phosphenium ion into the As1─As3 bond of **B_Mo_**, this bond is clearly broken in **4** (3.293(1) Å). Similarly, the As1–As3 distance in **5** (3.31(3) Å) indicates As─As bond fission. Notably, the remaining As─As bond distances (2.37(4) – 2.41(4) Å) correspond to slightly elongated As─As single bonds.^[^ ^63^ ^]^ While the spectroscopic data of **4** and **5** are less indicative compared to those of **1** and **2**, they manifest their purity. Intriguingly, the temperature sensitivity of **4** and **5** is far less pronounced, allowing their storage at room temperature in both solution and the solid state.

**Figure 4:**
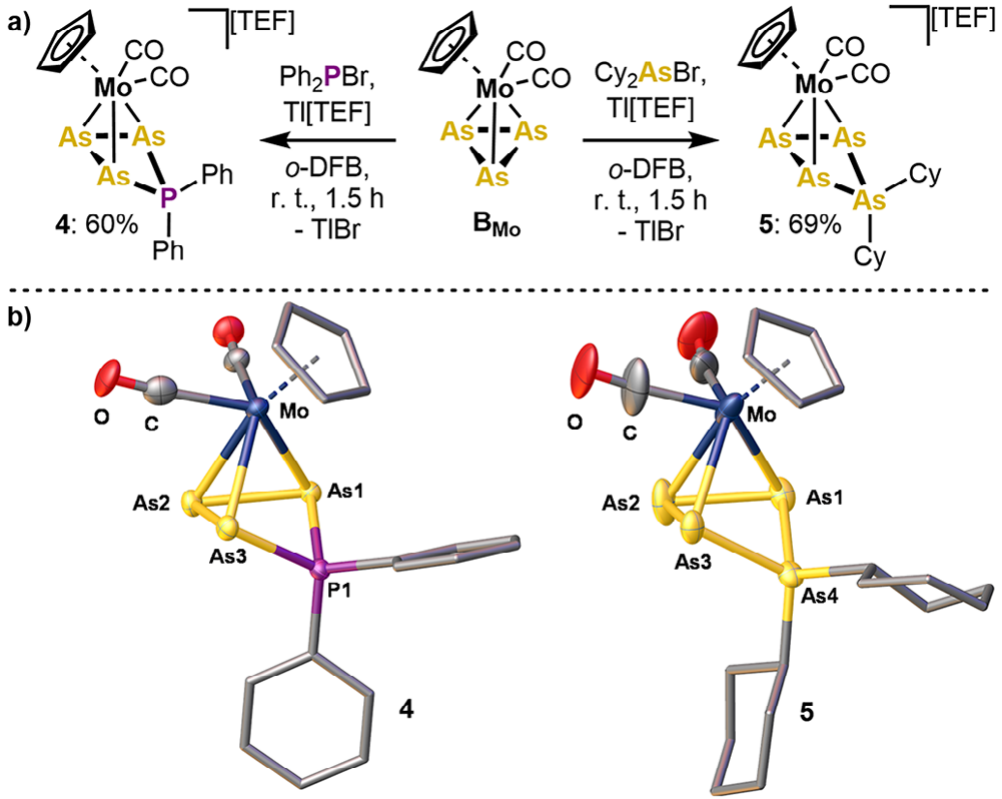
a) Synthesis of the *cyclo*‐As_3_PPh_2_ complex **4** and the *cyclo*‐As_4_Cy_2_ complex **5** via pnictogenium ion insertion into the *cyclo*‐As_3_ complex [{CpMo(CO)_2_}(η^3^‐As_3_)] (**B_Mo_**); b) molecular structures of **4** and **5** in the solid state with hydrogen atoms and the counter anion being omitted for clarity; anisotropic displacement parameters are drawn at the 50% probability level.

In summary, a synthetic approach has been developed, which overcomes the current limitation of arsenium ions to undergo bond insertion reactivity. Strain‐release driven arsenium ion bond insertion could be achieved utilizing highly strained *cyclo*‐Pn_3_ ligand complexes (Pn = P, As) as substrates. This provides the first structurally authenticated instance of such insertion reactivity being observed for arsenium ions. Utilizing this concept allowed for the preparation of the unprecedented *cyclo*‐P_3_AsCy_2_ complexes **1** and **2**, which showed remarkable temperature sensitivity. Quantum crystallographic investigation of the electronic structure of **1** and the computational elaboration of the mechanism affording **1** and **2** highlighted the importance of ring strain‐release for this synthetic approach. Additional reactivity studies involving the isolation of the arsenium coordinated **3** confirmed this assessment. Finally, the developed approach could be exploited to access an unprecedented *cyclo*‐As_4_Cy_2_ complex **5**, as well as its lighter homolog **4**, displaying unique representatives of cationic functionalization of an As_n_ ligand. Conclusively, the developed synthetic approach allows for the reliable insertion of arsenium ions into non‐polar bonds, based on the alleviation of ring strain in the substrate. Although demonstrated for *cyclo*‐Pn_3_ ligands (Pn = P, As) this approach is expected to be easily transferrable even to organic chemistry. Thus, exploiting the ring strain in small organic molecules, such as recently popularized BCBs or *cyclo*‐propanes could also allow for the insertion of arsenium ions into C─C bonds. Moreover, unlocking this fundamental mode of reactivity could display the initial step on the way toward arsenium ion redox catalysis.

## Supporting Information

The authors have cited additional references within the Supporting Information.^[^ ^51^, ^58^, ^59^, ^65^, ^66^, ^67^, ^68^, ^69^, ^70^, ^71^, ^72^, ^73^, ^74^, ^75^, ^76^, ^77^, ^78^, ^79^, ^80^, ^81^, ^82^, ^83^, ^84^, ^85^, ^86^, ^87^, ^88^, ^89^, ^90^, ^91^, ^92^, ^93^, ^94^, ^95^, ^96^, ^97^, ^98^ ^]^

Deposition Numbers CCDC‐2444647 (**1**, IAM), 2444379 (**3**), 2444380 (**4**), 2444381 (**5**), and 2441439 (**1**, HAR additional quantum crystallographic information is available under https://doi.org/10.5281/zenodo.15228936) contain the supplementary crystallographic data for this paper. These data are provided free of charge by the joint Cambridge Crystallographic Data Centre (https://www.ccdc.cam.ac.uk/services/structures?id=doi:10.1002/chem.202402675) and Fachinformationszentrum Karlsruhe (http://www.ccdc.cam.ac.uk/structures).

## Conflict of Interests

The authors declare no conflict of interest.

## Supporting information



Supporting Information

## Data Availability

The data that support the findings of this study are available in the Supporting Information of this article.
