# Supplementary material for: Strain‐Release Driven Arsenium Ion Bond Insertion
Source: Angew Chem Int Ed Engl. 2025 Jul 24;64(35):e202510186. doi: 10.1002/anie.202510186 (PMC12377436; doi:10.1002/anie.202510186)
Supplement: Supplementary file 1 — Supporting Information [file ANIE-64-e202510186-s001.pdf]

Strain-Release Driven Arsenium Ion Bond Insertion

Christoph Riesinger,* Florian Meurer, Lisa Zimmermann, Luis Dütsch, and Manfred Scheer*

Author Contributions

Christoph Riesinger – Conceptualization, synthesis and characterization of **1**, **2**, **4** and **5**, computational analysis, writing of original draft.

Florian Meurer – Quantum crystallographic analysis, writing of original draft.

Lisa Zimmermann – synthesis and characterization of compound **3**.

Luis Dütsch – crystallographic characterization of compound **4**.

Manfred Scheer – project administration, funding acquisition.

All authors contributed in preparing the final manuscript.

Synthesis and Analytical Data 3
NMR Spectra 9
X-ray Crystallography 18
Quantum Crystallography 24
Computational Details 32
References 42

Synthesis and Analytical Data

General Considerations

All manipulations were carried out using standard Schlenk techniques at a Stock apparatus under N_2 as an inert gas or in a glove box with Ar atmosphere. All glassware was dried with a heat gun (600 °C) for at least 30 min prior to use. *o*-DFB (1,2-difluorobenzene) was distilled from P_2O_5, CD_2Cl_2 was distilled from CaH_2 and other solvents were directly taken from an MBraun SPS-800 solvent purification system, degassed at room temperature and stored over molecular sieves. Solution ^1H (400.130 MHz), ^{19}F (376.498 MHz), and ^{31}P (161.976 MHz) NMR spectra were recorded at an Avance400 (Bruker) spectrometer using $(H_3C)_4Si$ (^1H, ^{13}C), $CFCl_3$ (^{19}F) or 85% phosphoric acid (^{31}P), respectively, as external standards. Chemical shifts (δ) are provided in parts per million (ppm) and coupling constants (J) are reported in Hertz (Hz). Chemical shifts and coupling constants for $^{31}P\{^1H\}$ and ^{31}P NMR spectra were partly derived from spectral simulation using the built-in simulation package of TopSpin3.2. The following abbreviations are used: s = singlet, d = doublet, dd = doublet of doublets, dt = doublet of triplets, t = triplet, td = triplet of doublets br = broad and m = multiplet. Mass spectra were recorded at the internal mass spectrometry department using a ThermoQuest Finnigan TSQ 7000 (ESI), Finnigan MAT 95 (LIFDI) mass spectrometer or by the first author on a Waters Micromass LCT ESI-TOF mass-spectrometer and peak assignment was performed using the Molecular weight calculator 6.50.[1] IR spectra were recorded as solids using a ThermoFisher Nicolet iS5 FT IR spectrometer with an iD7 ATR module and an ITX Germanium or ITX Diamond crystal. Elemental analysis of the products was conducted by the elemental analysis department at the University of Regensburg using an Elementar Vario EL. The starting materials [Cp'''Ni(η^3-P_3)],[2] [{CpMo(CO)$_2$}(η^3-P_3)],[3] [{CpMo(CO)$_2$}$_2$(μ,$\eta^{2:2}$-P_2)],[3] [{CpMo(CO)$_2$}(η^3-As_3)],[4] Tl[TEF],[5] Tl[BAr^{F24}][6] and Cy_2AsBr[7] were synthesized according to literature procedures. All other chemicals were purchased from commercial vendors and used without further purification.

*Synthesis of [Cp'''Ni(η^3-P_3AsCy$_2$)][TEF] (**1**)*

[Cp'''Ni(η^3-P_3)] (78 mg, 0.2 mmol, 1 eq.) and Cy_2AsBr (40 µL, exc.) were dissolved in 3 ml of *o*-DFB and Tl[TEF] (234 mg, 0.2 mmol, 1 eq.) dissolved in 4 mL of *o*-DFB was slowly added at -30 °C. A rapid color change to dark red and the precipitation of white solid (TlBr) were observed within seconds. The mixture was stirred at -30 °C for 3 h, then constrained to 2 mL and 40 mL of *n*-hexane were added to precipitate a red solid. The supernatant was decanted, the residue washed two times with 5 mL of *n*-hexane, each, and the solids were dried under reduced pressure (10^{-3} mbar, -30 °C). The red solid was dissolved in 3 mL of *o*-DFB, filtered and then directly layered with 60 mL of *n*-hexane. Storage of this mixture for one week at -30 °C yielded bright red crystalline [Cp'''Ni(η^3-P_3AsCy$_2$)][TEF] (**1**), which could be isolated after decanting the solvent and drying under reduced pressure. Compound **1** is temperature sensitive and needs to be handled, purified and stored below temperatures of -10 °C. Otherwise it decomposes rapidly in both solution and the solid state forming the triple-decker [{Cp'''Ni}$_2$(μ,$\eta^{3:3}$-P_3)]$^+$ as the only detectable side-product (see Figure S 5).[8]

Yield: 239 mg (0.15 mmol, 75%)

Elemental analysis: Calculated (%) for $C_{45}H_{51}O_4F_{36}AlP_3NiAs\cdot(C_6H_4F_2)_{0.5}$: C: 34.93, H: 3.24; found: C: 35.12, H: 3.64

Although it does not co-crystallize with **1**, traces of *o*-DFB are reproducibly found in the NMR (^{1}H, ^{19}F) spectra of this compound, which is a result of drying the compound at low temperatures.

^{1}H-NMR (CD_2Cl_2, 300 K): δ/ ppm = 1.30 (s, 9 H, 1,2,4-$^t\underline{Bu}_3C_5H_2$), 1.44 (s, 9 H, 1,2,4-$^t\underline{Bu}_3C_5H_2$),1.39 – 2.04 (several overlapping multiplets, 20 H, Cy), 2.87 (m (br), 2 H, Cy$_{(geminal)}$)), 5.92 (s, 2 H, 1,2,4-$^tBu_3C_5\underline{H}_2$)

^{31}P{^{1}H}-NMR (CD_2Cl_2, 300 K): δ/ ppm = 115.2 (d (br), $^1J_{PA-PM}$ = 298 Hz, 2 P, P^A), 91.6 (t (br), $^1J_{PA-PM}$ = 298 Hz, 1 P, P^M)

^{31}P-NMR (CD_2Cl_2, 300 K): δ/ ppm = 115.2 (d (br), $^1J_{PA-PM}$ = 298 Hz, 2 P, P^A), 91.6 (t (br), $^1J_{PA-PM}$ = 298 Hz, 1 P, P^M)

^{19}F{^{1}H}-NMR (CD_2Cl_2, 300 K): δ/ ppm = -75.59 (s, [TEF]$^-$)

ESI(+)-MS (*o*-DFB): *m/z* (%) = 499.2 (100, [$(Cy_2As)_2OH$]$^+$), 625.2 (90, [**1**]$^+$)

*Synthesis of [{CpMo(CO)$_2$}(η^3-P$_3$AsCy$_2$)][TEF] (**2**)*

[{CpMo(CO)$_2$}(η^3-P$_3$)] (31 mg, 0.1 mmol, 1 eq.) and Cy$_2$AsBr (20 µL, exc.) were dissolved in 3 ml of *o*-DFB and Tl[TEF] (117 mg, 0.1 mmol, 1 eq.) dissolved in 4 mL of *o*-DFB was slowly added at -30 °C. A rapid color change from yellow to dark yellowish/orange and the precipitation of white solid (TlBr) were observed within seconds. The mixture was stirred at 0 °C for 3 h, then constrained to 2 mL and 40 mL of *n*-hexane (0 °C) were added to precipitate an orange solid. The supernatant was decanted, the residue washed two times with 5 mL of *n*-hexane, each, and the solids were dried under reduced pressure (10^{-3} mbar). The orange solid was dissolved in 3 mL of *o*-DFB (0 °C), filtered and then 60 mL of *n*-hexane (0 °C) were added to precipitate [{CpMo(CO)$_2$}(η^3-P$_3$AsCy$_2$)][TEF] (**2**) as an orange solid, which was dried under reduced pressure (10^{-3}mbar) at 0 °C.

Yield: 129 mg (0.085 mmol, 85%)

Elemental analysis: Calculated (%) for C$_{35}$H$_{27}$O$_6$F$_{36}$AlP$_3$AsMo: C: 27.69, H: 1.79; found: C: 28.05, H: 1.95

^{1}H-NMR (o-DFB/C$_6$D$_6$, 300 K): δ/ ppm = 1.0 – 2.1 (several overlapping multiplets, 20 H, Cy), 2.31 (m, 1 H, Cy$_{(geminal)}$), 2.69 (m, 1 H, Cy$_{(geminal)}$), 5.82 (s, 5 H, Cp)

^{31}P{^{1}H}-NMR (o-DFB/C$_6$D$_6$, 300 K): δ/ ppm = 67.7 (d, $^1J_{PA-PM}$ = 280 Hz, 2 P, P^A), -58.0 (d, $^1J_{PA-PM}$ = 280 Hz, 1 P, P^M)

^{31}P-NMR (o-DFB/C$_6$D$_6$, 300 K): δ/ ppm = 67.7 (d, $^1J_{PA-PM}$ = 280 Hz, 2 P, P^A), -58.0 (d, $^1J_{PA-PM}$ = 280 Hz, 1 P, P^M)

^{19}F{^{1}H}-NMR (o-DFB/C$_6$D$_6$, 300 K): δ/ ppm = -75.45 (s, [TEF]$^-$)

ESI(+)-MS (*o*-DFB): *m/z* (%) = 483.0 (50, [(Cy$_2$As)$_2$H]$^+$), 499.2 (100, [(Cy$_2$As)$_2$OH]$^+$), 524.9 (15, [**2**-CO]$^+$), 541.9 (20, [(**2**-C)+H]$^+$), 552.9 (8, [**2**]$^+$), 723.2 (40, unknown aggregation/fragmentation product), 783.0 (8, unknown aggregation/fragmentation product), 832.7 (10, [(**2**-CO)+**A$_{Mo}$**]$^+$)

IR: $\tilde{\nu}$(CO)/cm^{-1} = 2063 (m), 2021 (m)

*Synthesis of [{CpMo(CO)$_2$}$_2$(μ,$\eta^{2:2}$-P$_2$AsCy$_2$)][TEF] (**3**)*

Cy_2AsBr (15 µL, 0.1 mmol, 1 eq.) in *o*-DFB (1 mL) was added slowly to a red solution of [{CpMo(CO)$_2$}$_2$(µ,$\eta^{2:2}$-P$_2$)] (50 mg, 0.1 mmol, 1 eq.) and Tl[TEF] (117 mg, 0.1 mmol, 1 eq.) in *o*-DFB (2 mL). Immediate precipitation of white solid (TlBr) and a colour change to dark red was observed. After stirring for 3 h at room temperature, the solvent was removed *in vacuo* and the precipitate was washed twice with *n*-hexane (10 mL each). After drying under reduced pressure (10^{-3} mbar), the residue was dissolved in *o*-DFB (3 mL), filtered through a glass fiber filter paper and layered with *n*-hexane (20 mL), Storage for 14 days at room temperature yielded red blocks of [{CpMo(CO)$_2$}$_2$(µ,$\eta^{2:2}$-P$_2$AsCy$_2$)][TEF] (**3**) suitable for single crystal X-ray analysis.

Yield: 60 mg (0.039 mmol, 39%).

Elemental Analysis: calc. (%) for $C_{42}H_{32}O_8F_{36}AlP_2AsMo_2$: C: 29.60, H: 1.89.

found (%): C: 30.05, H: 1.79.

^{1}H-NMR (CD_2Cl_2,300 K): δ/ ppm = 1.32-2.35 (several broad overlapping multiplets, 22 H, Cy), 5.64 (s, 10 H, Cp).

^{31}P{^{1}H}-NMR (CD_2Cl_2, 300 K): δ/ ppm = -122.4 (s(*br*), 1 P, P-P-AsCy$_2$), -79.0 (s(*br*), 1 P, P-P-AsCy$_2$).

^{31}P-NMR (CD_2Cl_2, 300 K): δ/ ppm = -122.4 (s(*br*), 1 P, P-P-AsCy$_2$), -79.0 (s(*br*), 1 P, P-P-AsCy$_2$).

^{19}F{^{1}H}-NMR (CD_2Cl_2, 300 K): δ/ ppm = -75.71 (s(, 36 F, [TEF]$^-$).

ESI(+) MS (*o*-DFB): *m/z* (%) = 737.22 (100%, [**3**]$^+$ (M$^+$)), 496.10 (15%, [Mo$_2$P$_2$]$^+$), 282.35 (20%, *unidentified*), 565.57 (30%, *unidentified*).

IR: $\tilde{\nu}$(CO)/cm^{-1} = 2046 (m), 2008 (m), 1984 (m), 1940 (m).

*Synthesis of [{CpMo(CO)$_2$}(η^3-As$_3$PPh$_2$)][TEF] (**4**)*

[{CpMo(CO)$_2$}(η^3-As$_3$)] (45 mg, 0.1 mmol, 1 eq.) and Ph_2PCl (18 μL, 0.1 mmol, 1 eq.) were dissolved in 2 mL of *o*-DFB and Tl[TEF] (117 mg, 0.1 mmol, 1 eq.) dissolved in 2 mL of *o*-DFB was slowly added to this mixture at room temperature. Precipitation of a colorless solid occurred within seconds. After stirring the solution for 1.5 h at room temperature it was separated from the solids via filtration. Addition of 50 mL of *n*-hexane precipitated [{CpMo(CO)$_2$}(η^3-As$_3$PPh$_2$)][TEF] (**4**) as a light yellowish solid, which could be isolated after decanting the solvent and drying under reduced pressure (10^{-3} mbar). **4** could be recrystallized by layering a concentrated solution in *o*-DFB with *n*-hexane and storage at room temperature for one week.

**Yield**:	96 mg (0.06 mmol, 60%)
**Elemental analysis**:	Calculated (%) for $C_{35}H_{15}O_6F_{36}AlPAs_3Mo$: C: 26.37, H: 0.95; found: C: 26.95, H: 1.21
**^{1}H-NMR** (CD_2Cl_2, 300 K):	δ/ ppm = 5.71 (s, 5 H, Cp), 7.35 (m, 2 H, Ph), 7.68 (m (overlapping), 3 H, Ph), 7.78 (m, 2 H, Ph), 7.86 (m (overlapping), 3 H, Ph)
**^{31}P{^{1}H}-NMR** (CD_2Cl_2, 300 K):	δ/ ppm = 0.27 (s, PPh_2)
**^{31}P-NMR** (CD_2Cl_2, 300 K):	δ/ ppm = 0.27 (m, PPh_2
**^{19}F{^{1}H}-NMR** (CD_2Cl_2, 300 K):	δ/ ppm = -75.61 (s, $[TEF]^-$)
**ESI(+)-MS** (*o*-DFB):	*m/z* (%) = 628.75 (100%, [**4**]$^+$)
**IR**:	$\tilde{\nu}$(CO)/cm^{-1} = 2038 (s), 2001 (s)

*Synthesis of [{CpMo(CO)$_2$}(η^3-As$_4$Cy$_2$)][TEF] (**5**)*

[{CpMo(CO)$_2$}(η^3-As$_3$)] (16 mg, 0.035 mmol, 1 eq.) and Cy$_2$AsBr (20 µL, exc.) were dissolved in 3 mL of *o*-DFB and Tl[TEF] (41 mg, 0.035 mmol, 1 eq.) dissolved in 2 mL of *o*-DFB was slowly added to this mixture at room temperature and under the exclusion of light. Precipitation of a colorless solid occurred within seconds. After stirring the solution for 1.5 h at room temperature it was constrained to 2 mL and then separated from the solids via filtration. Removal of the solvent under reduced pressure (10^{-3}mbar) afforded [{CpMo(CO)$_2$}(η^3-As$_4$Cy$_2$][TEF] (**5**) as a bright yellow solid. **5** could be recrystallized by layering a concentrated solution in CH_2Cl_2 with *n*-hexane and storage at room temperature for one week.

**Yield**:	40 mg (0.024 mmol, 69%)
**Elemental analysis**:	Calculated (%) for $C_{35}H_{27}O_6F_{36}AlAs_4Mo\ (C_6H_{14})_{0.25}$:<br>C: 26.22, H: 1.84; found: C: 26.23, H: 1.87<br><br>Traces of *n*-hexane are also found in the ^{1}H NMR spectra of **5**.
**^{1}H-NMR** (CD_2Cl_2, 300 K):	δ/ ppm = 1.15 – 2.14 (several overlapping multiplets, 22 H, Cy), 2.35 (m, 1 H, Cy$_{(geminal)}$), 2.70 (m, 1 H, Cy$_{(geminal)}$), 6.07 (s, 5 H, Cp)
**^{19}F{^{1}H}-NMR** (CD_2Cl_2, 300 K):	δ/ ppm = -75.61 (s, [TEF]$^-$)
**ESI(+)-MS** (*o*-DFB):	*m/z* (%) = 499.2 (80, [(Cy$_2$As)$_2$OH]$^+$), 684.8 (100, [**5**]$^+$)
**IR**:	$\tilde{\nu}$(CO)/cm^{-1} = 2040 (m), 1993 (m)

NMR Spectra

*[Cp'''Ni(η^3-P_3AsCy_2)][TEF] (**1**)*

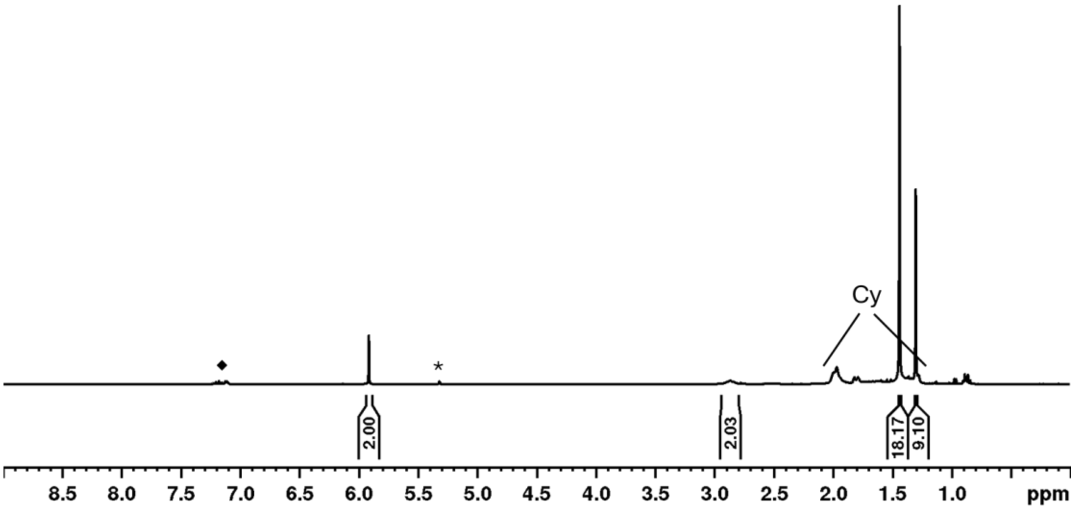

*Figure S 1: ^{1}H NMR spectrum of **1** in CD_2Cl_2 recorded at room temperature; ◆ marks the signal of residual o-DFB in the product and * marks the solvent residual signal of CD_2Cl_2.*

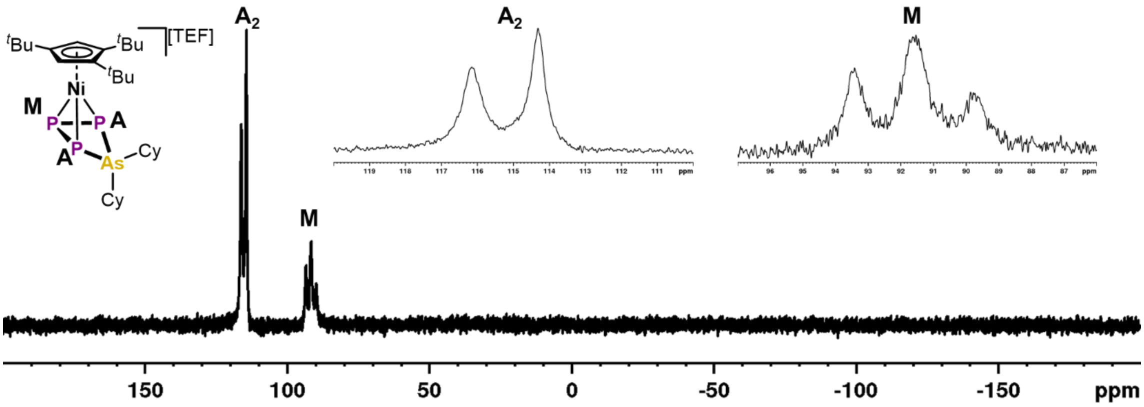

*Figure S 2: ^{31}P{^{1}H} NMR spectrum of **1** in CD_2Cl_2 recorded at room temperature; signal assignment according to the inlet of the molecular structure of **1**.*

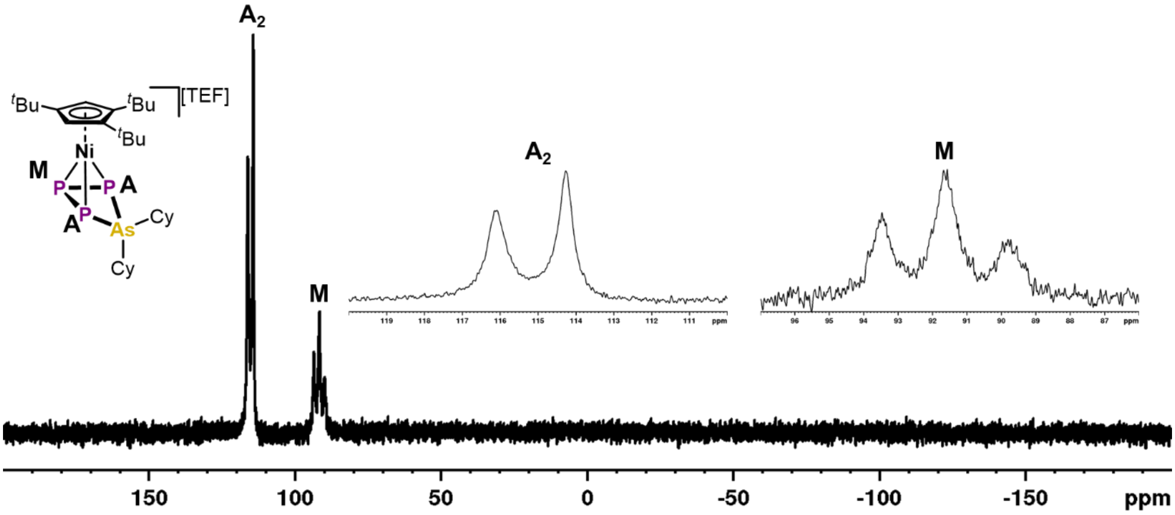

*Figure S 3: ^{31}P NMR spectrum of **1** in CD_2Cl_2 recorded at room temperature; signal assignment according to the inlet of the molecular structure of **1**.*

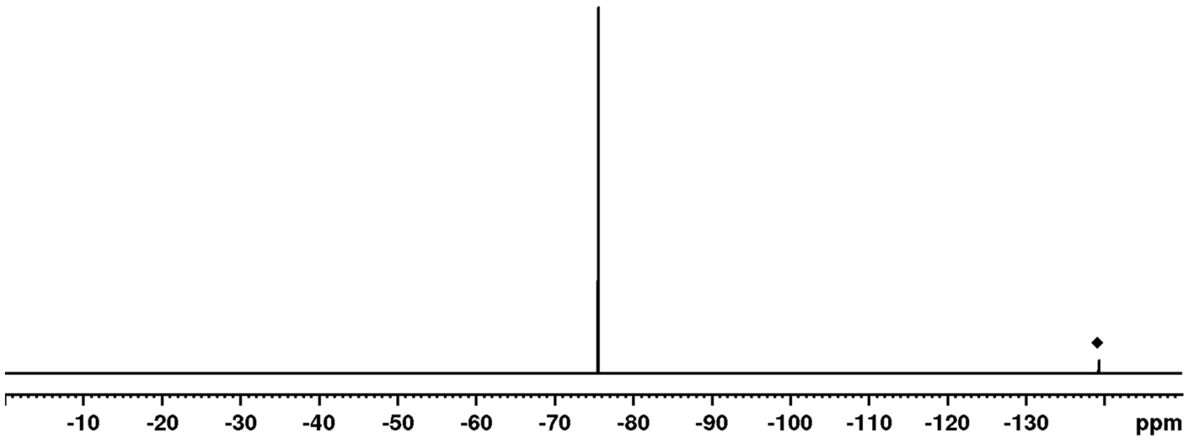

*Figure S 4: $^{19}F\{^{1}H\}$ NMR spectrum of **1** in CD_2Cl_2 recorded at room temperature; ◆ marks the signal of residual o-DFB.*

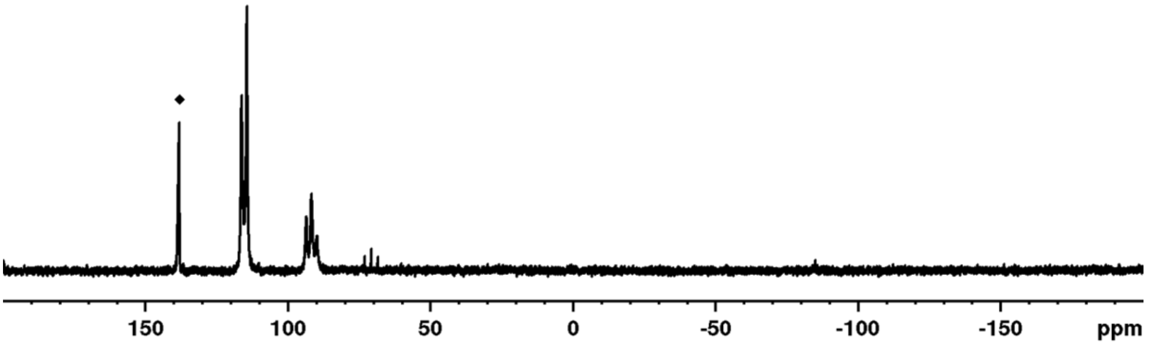

*Figure S 5: $^{31}P\{^{1}H\}$ NMR spectrum of **1** in o-DFB (with C_6D_6 capillary) recorded 4 h after it was dissolved and kept at room temperature; ◆ marks the signal of $[\{Cp'''Ni\}_2(\mu,\eta^{3:3}\text{-}P_3)]^+$, the main decomposition product of **1**.*

$[\{CpMo(CO)_2\}(\eta^3\text{-}P_3AsCy_2)][BAr^{F24}]$ (**2**)

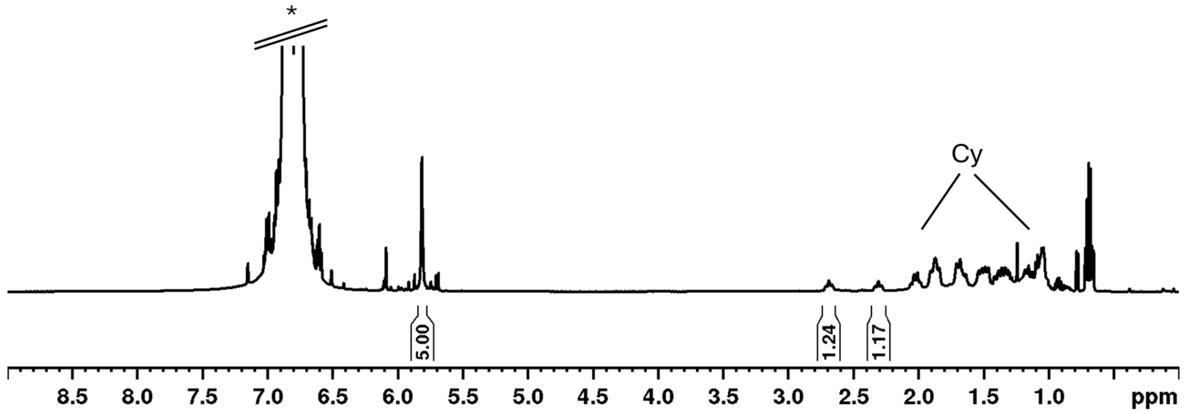

*Figure S 6: ^{1}H NMR spectrum of **2** in CD_2Cl_2 recorded at room temperature; * marks the solvent signal of o-DFB; 2 decomposes rapidly in CD_2Cl_2.*

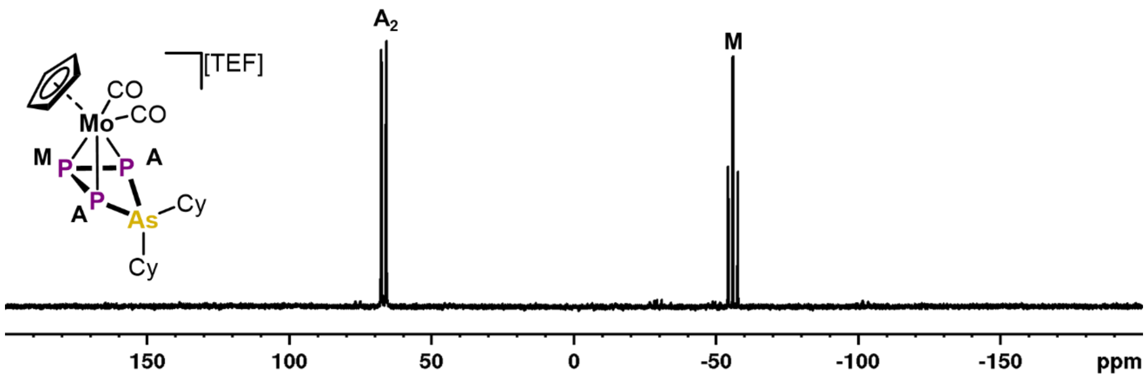

*Figure S 7: $^{31}P\{^1H\}$ NMR spectrum of **2** in CD_2Cl_2 recorded at room temperature.*

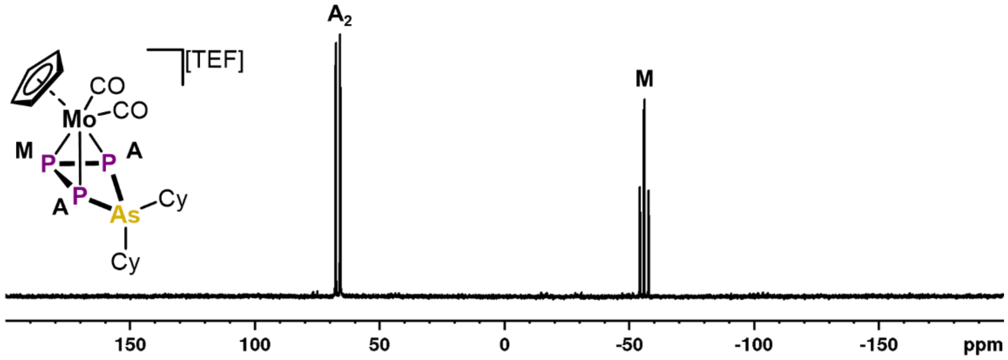

*Figure S 8: ^{31}P NMR spectrum of **2** in CD_2Cl_2 recorded at room temperature.*

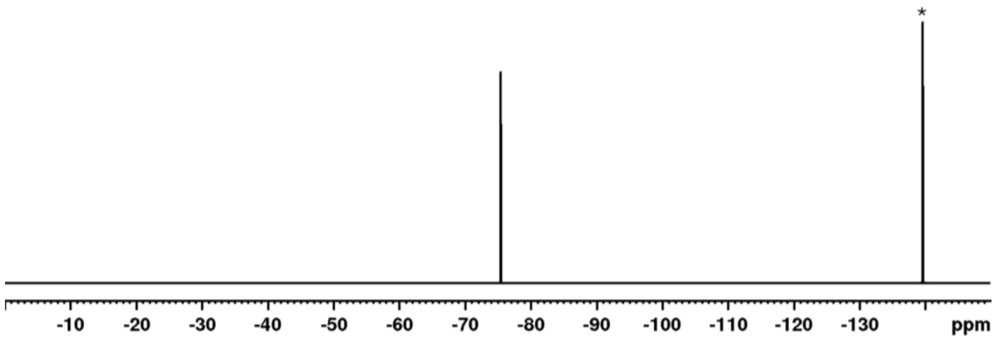

*Figure S 9: $^{19}F\{^1H\}$ NMR spectrum of **2** in CD_2Cl_2 recorded at room temperature; * marks the solvent signal of o-DFB.*

When the Cp ligand in **2** is replaced with the sterically much more demanding Cp* (= C_5Me_5) ligand in **2*** ($[BAr^{F24}]^-$ = $[B\{3,5-(CF_3)_2-C_6H_3\}_4]^-$), the insertion of the arsenium ion is revoked. This effect can easily be observed by comparing the $^{31}P\{^1H\}$ NMR spectra of the respective compound in CD_2Cl_2 (Figure S10). While **2** shows the expected A_2M spin system (*vide supra*) in line with arsenium ion insertion, **2*** only yields a broad signal centered at δ/ ppm = 306. The latter is only slightly shifted compared to the starting material,[9] clearly indicating that the sterically demanding Cp* ligand inhibits arsenium ion insertion and thus only leads to its coordinative stabilization.

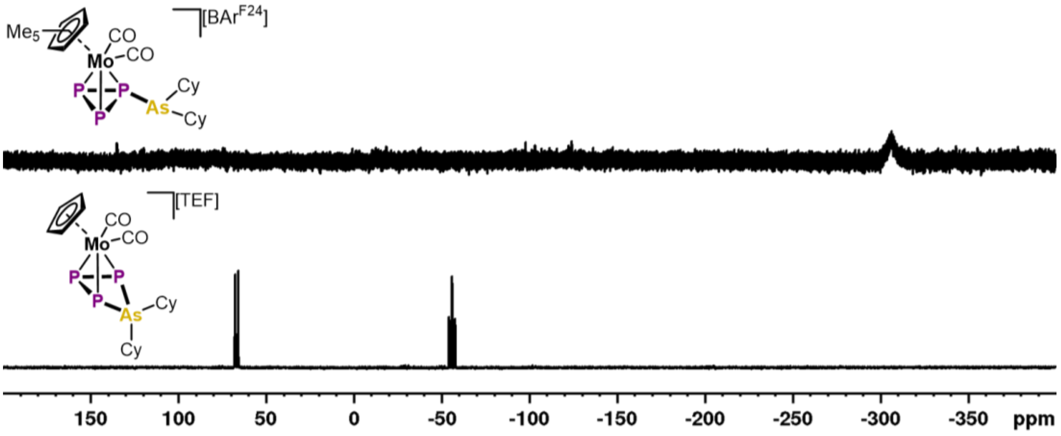

*Figure S 10: Experimental $^{31}P\{^1H\}$ NMR spectra of **2** (bottom) and its Cp* derivative **2*** (top) in CD_2Cl_2 recorded at room temperature.*

*[{CpMo(CO)$_2$}$_2$(μ,$\eta^{2:2}$-P$_2$AsCy$_2$)][TEF] (**3**)*

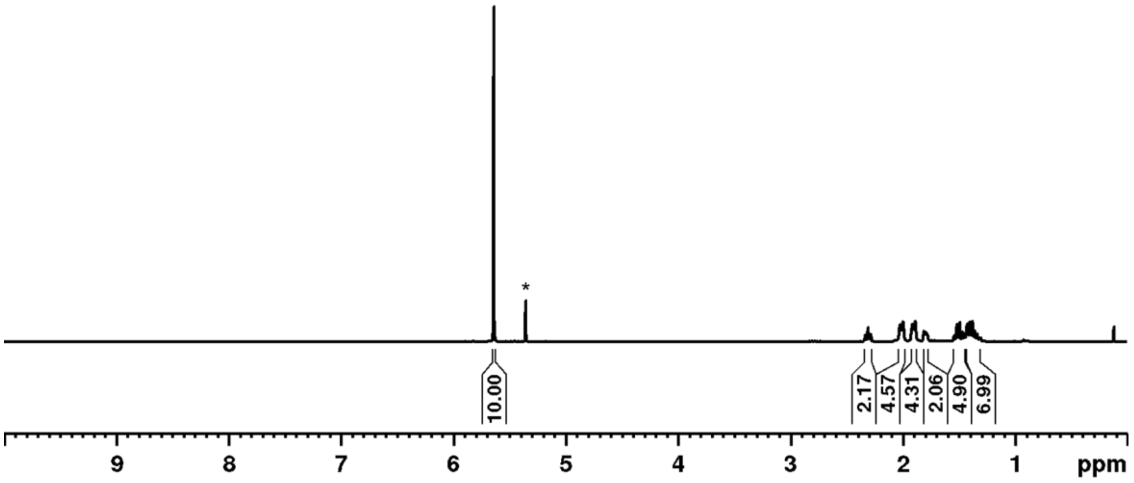

*Figure S 11: ^{1}H NMR spectrum of **3** in CD2Cl2 at room temperature. * marks residual solvent signal of CD_2Cl_2.*

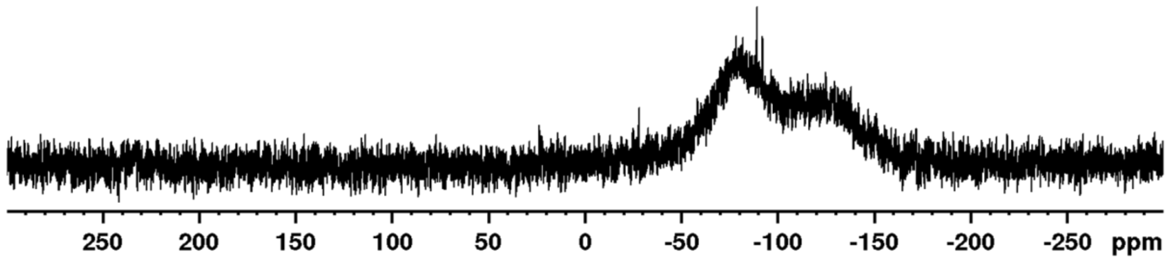

*Figure S 12: ^{31}P{^{1}H} NMR spectrum of **3** in CD_2Cl_2 at room temperature.*

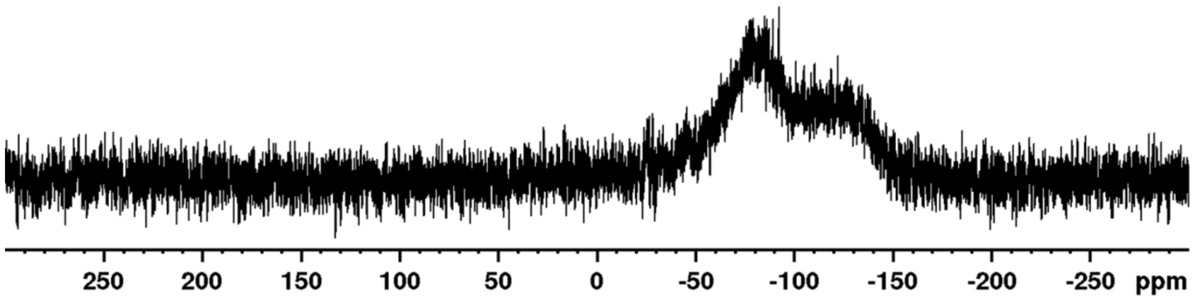

*Figure S 13: ^{31}P NMR spectrum of **3** in CD_2Cl_2 at room temperature.*

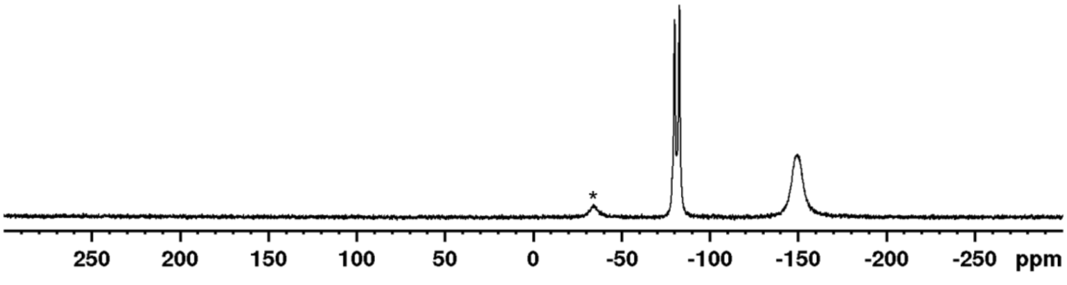

*Figure S 14: ^{31}P{^{1}H} NMR spectrum of **3** in CD_2Cl_2 at 193 K. * = [{CpMo(CO)$_2$}$_2$(μ,$\eta^{2:2}$-P$_2$)].*

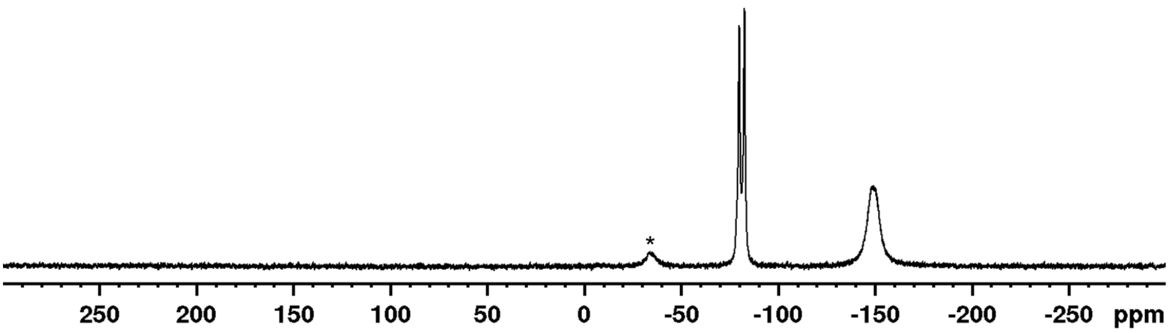

*Figure S 15: ^{31}P NMR spectrum of **3** in CD_2Cl_2 at 193 K. * = [{CpMo(CO)$_2$}$_2$($\mu,\eta^{2:2}$-P_2)].*

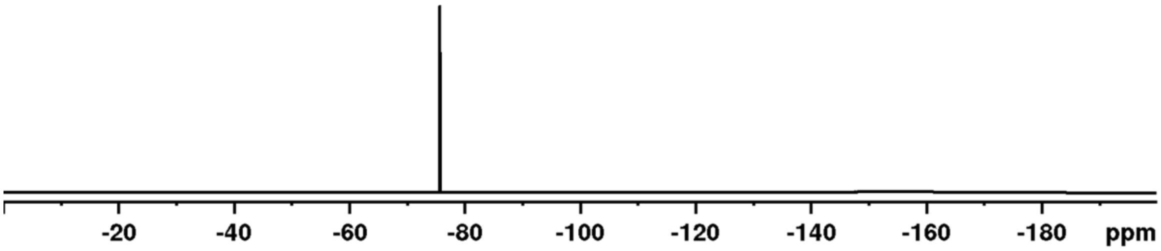

*Figure S 16: ^{19}F NMR spectrum of **3** in CD_2Cl_2 at room temperature.*

[{CpMo(CO)$_2$}(η^3-As$_3$PPh$_2$)][TEF] (**4**)

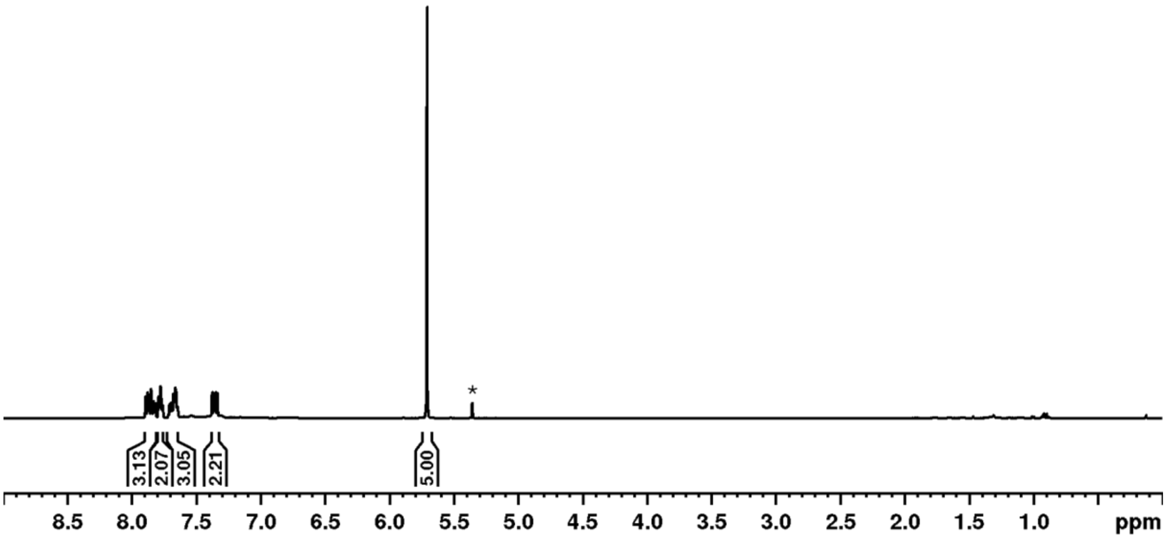

*Figure S 17: ^{1}H NMR spectrum of **4** in CD$_2$Cl$_2$ recorded at room temperature; * marks the solvent residual signal of CD$_2$Cl$_2$.*

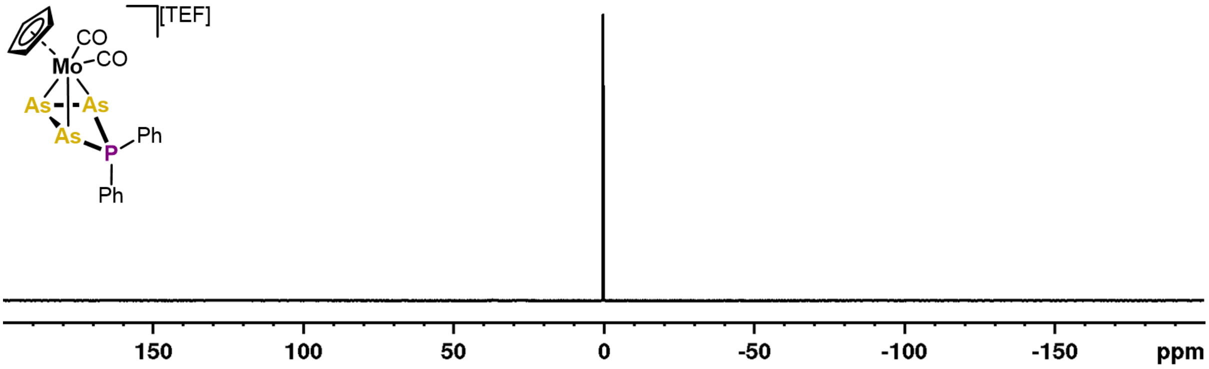

*Figure S 18: ^{31}P{^{1}H} NMR spectrum of **4** in CD$_2$Cl$_2$ recorded at room temperature.*

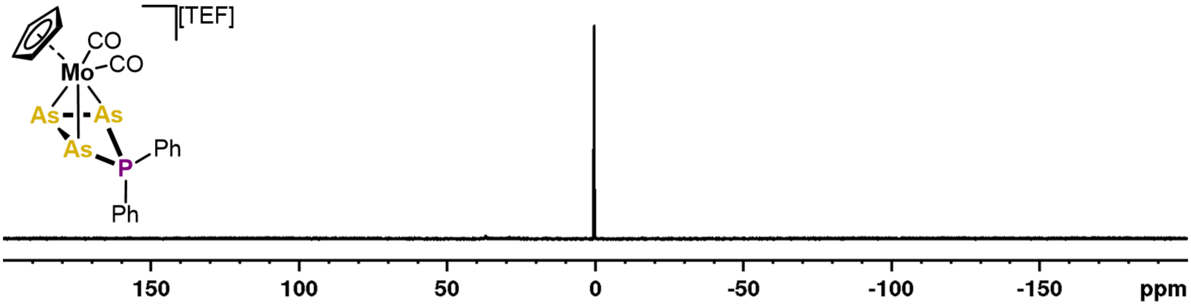

*Figure S 19: ^{31}P NMR spectrum of **4** in CD$_2$Cl$_2$ recorded at room temperature.*

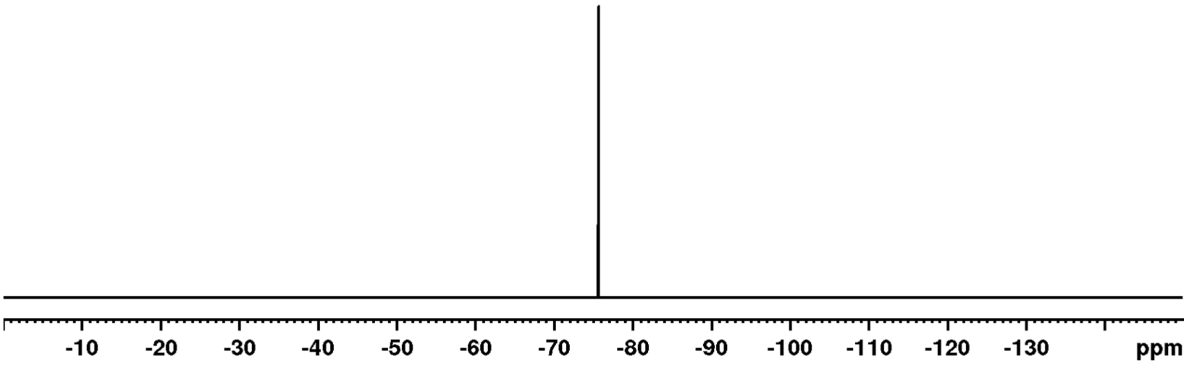

*Figure S 20: $^{19}F\{^{1}H\}$ NMR spectrum of **4** in CD_2Cl_2 recorded at room temperature.*

*[{CpMo(CO)$_2$}(η^3-As$_4$Cy$_2$)][TEF] (**5**)*

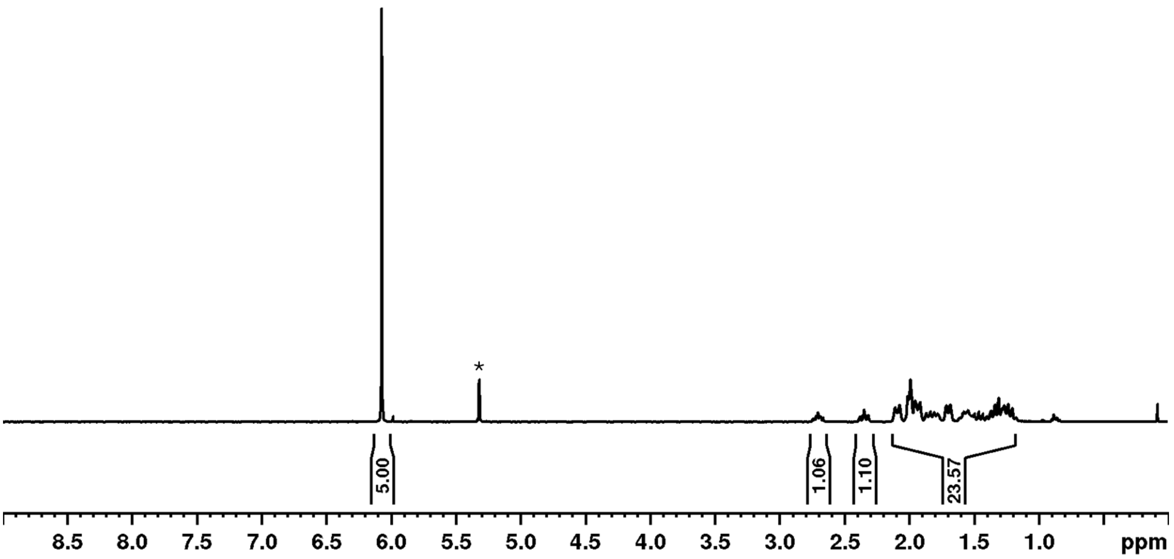

*Figure S 21: ^{1}H NMR spectrum of **5** in CD_2Cl_2 recorded at room temperature; * marks the solvent residual signal of CD_2Cl_2; the integral for of the cyclohexyl groups is slightly overestimated (23.6 instead of the expected 20), which is due to overlap with signals assigned to n-hexane.*

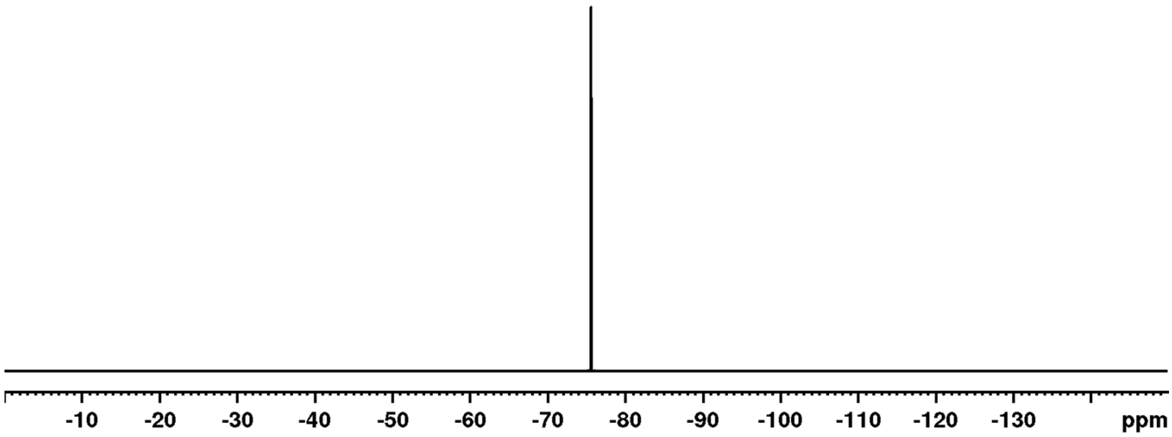

*Figure S 22: ^{19}F{^{1}H} NMR spectrum of **5** in CD_2Cl_2 recorded at room temperature.*

X-ray Crystallography

General considerations

The crystallographic data for all synthesized compounds was collected either on an Xcalibur Gemini with an AtlasS2 detector using Cu-K_α radiation (**4**), or on a XtaLAB Synergy R, DW system with HyPix-Arc 150 detector applying Cu-Kα radiation (**1**, **3**, **5**) from a rotating anode X-ray source. All measurements were performed at 123 K. Data collection, data reduction and absorption correction were performed with the CrysAlisPro software package.[10] Structure solution and refinement was conducted in Olex2[11] with ShelXT[12] and ShelXL[13] (full-matric least-squares method against F^2). All non-hydrogen atoms were refined with anisotropic displacement parameters and hydrogen atoms were treated as riding models with isotropic displacement parameters and fixed C-H bond lengths (sp^3: 0.96 (CH_3), 0.97 (CH_2), sp^2: 0.93 (CH)). Visualization of the crystal structures was performed with Olex2.[11]

CCDC entries CCDC- 2444647 (**1**, IAM), 2444379 (**3**), 2444380 (**4**), 2444381 (**5**), and 2441439 (**1**, HAR) contain the supplementary crystallographic data for this paper. These data can be obtained free of charge at www.ccdc.cam.ac.uk/conts/retrieving.html (or from the Cambridge Crystallographic Data Centre, 12 Union Road, Cambridge CB2 1EZ, UK; Fax: + 44-1223-336-033; e-mail: deposit@ccdc.cam.ac.uk).

Compound	1	3	4	5	A_{Ni}*
**Dataset Parameters and Information**					
Empirical formula	$C_{45}H_{51}O_4F_{36}AlP_3NiAs$	$C_{42}H_{32}AlAsF_{36}Mo_2O_8P_2$	$C_{35}H_{15}O_6F_{36}AlPAs_3Mo$	$C_{35}H_{26}AlAs_4F_{36}MoO_6$	$C_{17}H_{29}NiP_3$
Formula weight	1593.38	1704.39	1594.12	1649.16	356.81
Temperature/K	100.01(10)	123.15	293(2)	123.00(10)	100.15
Crystal system	triclinic	triclinic	orthorhombic	monoclinic	Monoclinic
Space group	$P\bar{1}$	$P\bar{1}$	$Pna2_1$	Pn	$P2_1/c$
a/Å	14.3555(3)	11.58340(10)	16.8787(4)	11.57440(10)	9.2644(1)
b/Å	14.7878(3)	21.22370(10)	26.5532(6)	16.3726(2)	14.5889(1)
c/Å	15.6447(4)	23.59390(10)	11.0660(2)	27.7049(3)	14.5261(1)
α/°	89.398(2)	91.1040(10)	90	90	90
β/°	68.150(2)	95.6360(10)	90	90.0630(10)	105.935(1)
γ/°	81.240(2)	91.4550(10)	90	90	90
Volume/Å^3	3042.75(13)	5769.10(6)	4959.60(19)	5250.16(10)	1887.87(3)
Z	2	4	4	4	4
ρ_{calc}g/cm^3	1.739	1.962	2.135	2.086	1.255
μ/mm^{-1}	3.361	6.422	6.571	6.620	1.270
F(000)	1592.0	3328.0	3064.0	3180.0	706.9
Crystal size/mm^3	0.38 × 0.18 × 0.12	0.32 × 0.22 × 0.18	0.705 × 0.248 × 0.241	0.17 × 0.14 × 0.09	0.29 × 0.27 × 0.23
Radiation	Cu $K_α$ (λ = 1.54184)	Cu $K_α$ (λ = 1.54184)	Cu $K_α$ (λ = 1.54184)	Cu $K_α$ (λ = 1.54184)	Mo $K_α$ (0.71073)
2Θ range /°	6.054 to 148.344	3.764 to 147.904	8.474 to 144.012	5.398 to 150.456	5.36 to 82.32
Index ranges	-15 ≤ h ≤ 17, -15 ≤ k ≤ 18, -19 ≤ l ≤ 19	-14 ≤ h ≤ 11, -26 ≤ k ≤ 26, -29 ≤ l ≤ 28	-20 ≤ h ≤ 20, -32 ≤ k ≤ 29, -13 ≤ l ≤ 13	-14 ≤ h ≤ 12, -19 ≤ k ≤ 20, -33 ≤ l ≤ 34	-17 ≤ h ≤ 17, -26 ≤ k ≤ 26, -26 ≤ l ≤ 26
Reflections collected	36395	189054	29084	50568	279297
Independent reflections	11721	22268	9326	18163	12436
R_{int}	0.0557	0.0375	0.0429	0.0248	0.0293
R_{sigma}	0.0368	0.0133	0.0331	0.0248	0.0089
**Model and Refinement Quality Indicators (IAM)**					
CCDC Nr.	2444647	2444379	2444380	2444381	-
Data/restr./param.	11721/30/932	22268/3462/2648	9326/247/831	18163/4105/2566	-
Goodness-of-fit on F^2	1.066	1.051	1.021	1.052	-
Final R_1 [I>=2σ (I)]	0.0515	0.0387	0.0384	0.1244	-
Final R_1 [all data]	0.0552	0.0395	0.0387	0.1244	-
Final wR_2 [I>=2σ (I)]]	0.1470	0.0980	0.1034	0.2890	-
Final wR_2 [all data]	0.1505	0.0985	0.1039	0.2895	-
Largest diff. peak/hole / e Å^{-3}	1.20/-1.63	1.30/-1.12	0.93/-1.56	1.56/-1.60	-
Flack parameter	-	-	0.476(13)	0.23(4)	-
**Quantum Crystallographic Quality Indicators (HAR)**					
CCDC Nr.	2441439†	-	-	-	2305141
Data/restr./param.	11711/717/1396	-	-	-	12436/0/537
Goodness-of-fit on F^2	1.050	-	-	-	1.059
Final R_1 [I>=2σ (I)]	0.0479	-	-	-	0.0114
Final R_1 [all data]	0.0515	-	-	-	0.0137
Final wR_2 [I>=2σ (I)]]	0.1345	-	-	-	0.0216
Final wR_2 [all data]	0.1379	-	-	-	0.0221
Largest diff. peak/hole / e Å^{-3}	1.02/-1.57	-	-	-	0.16/-0.18

* Data for $\mathbf{A}_{Ni}$ are from [16], model was re-refined in a HAR procedure employing the same settings as for **1**; † additional quantum crystallographic information, such as wave functions and the table of scatterers, is available under https://doi.org/10.5281/zenodo.15228936.

*[Cp'''Ni(η^3-P_3AsCy$_2$)][TEF] (**1**)*

1 crystallizes as bright red blocks from concentrated *o*-DFB solutions layered with *n*-hexane and storage at -30 °C for seven days (Figure S 27). **1** crystallizes in the monoclinic space group $P\bar{1}$ with one cation and one anion in the asymmetric unit. Disorder was treated with adequate restraints.

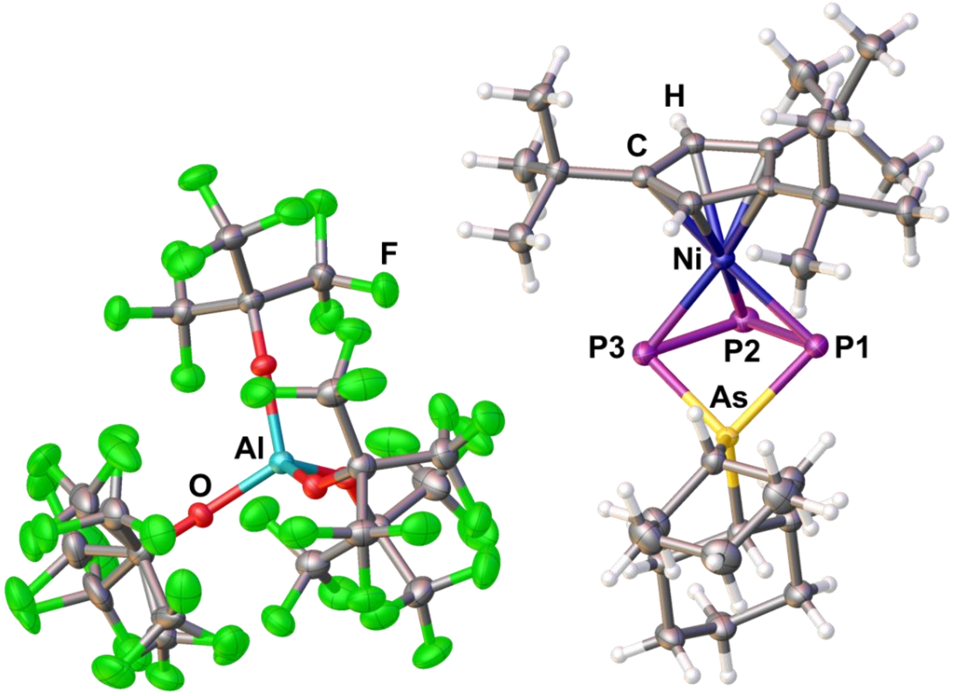

*Figure S 23: Solid state structure of **1**; Depicted is the asymmetric unit and ADPs (anisotropic displacement parameters) are drawn at 50 % probability.*

*[{CpMo(CO)$_2$}$_2$(μ,$\eta^{2:2}$-P$_2$AsCy$_2$)][TEF] (**3**)*

3 crystallizes as red block shaped crystals from concentrated *o*-DFB solutions layered with *n*-hexane and storage at room temperature for 14 days (Figure S 28). **3** crystallizes in the triclinic space group $P\bar{1}$ with two cations and two anions in the asymmetric unit. Disorder was treated with adequate restraints.

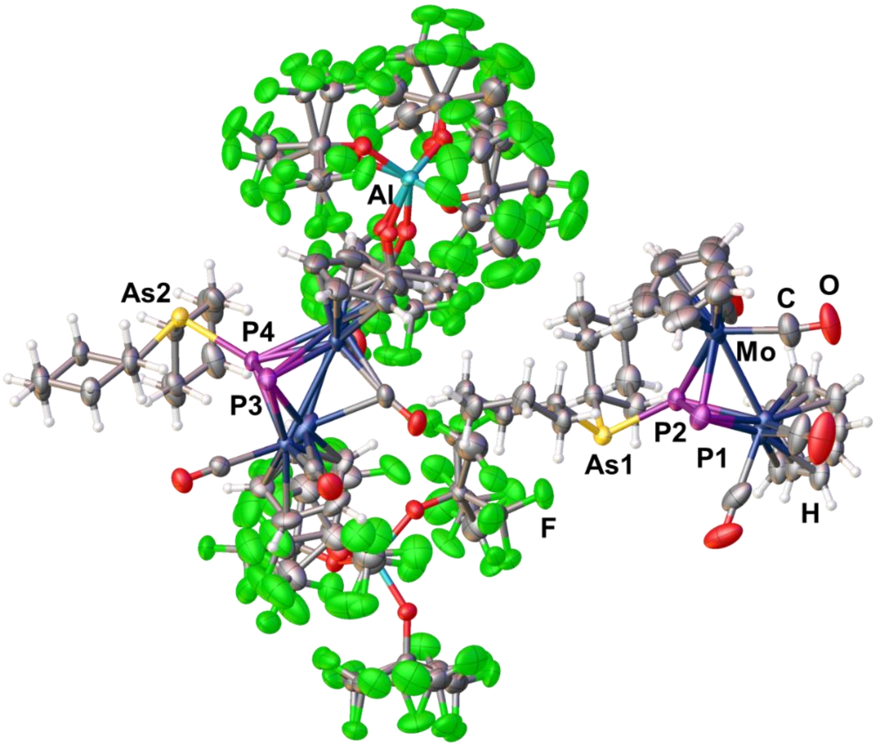

*Figure S 24 Solid state structure of **3**; Depicted is the asymmetric unit and ADPs (anisotropic displacement parameters) are drawn at 50 % probability.*

*[{CpMo(CO)$_2$}(η^3-As$_3$PPh$_2$)][TEF] (**4**)*

4 crystallizes as bright yellow needles from concentrated *o*-DFB solutions layered with *n*-hexane and storage at room temperature for seven days (Figure S 29). **4** crystallizes in the orthorhombic space group *Pna*2$_1$ with one cation and one anion in the asymmetric unit. Disorder was treated with adequate restraints.

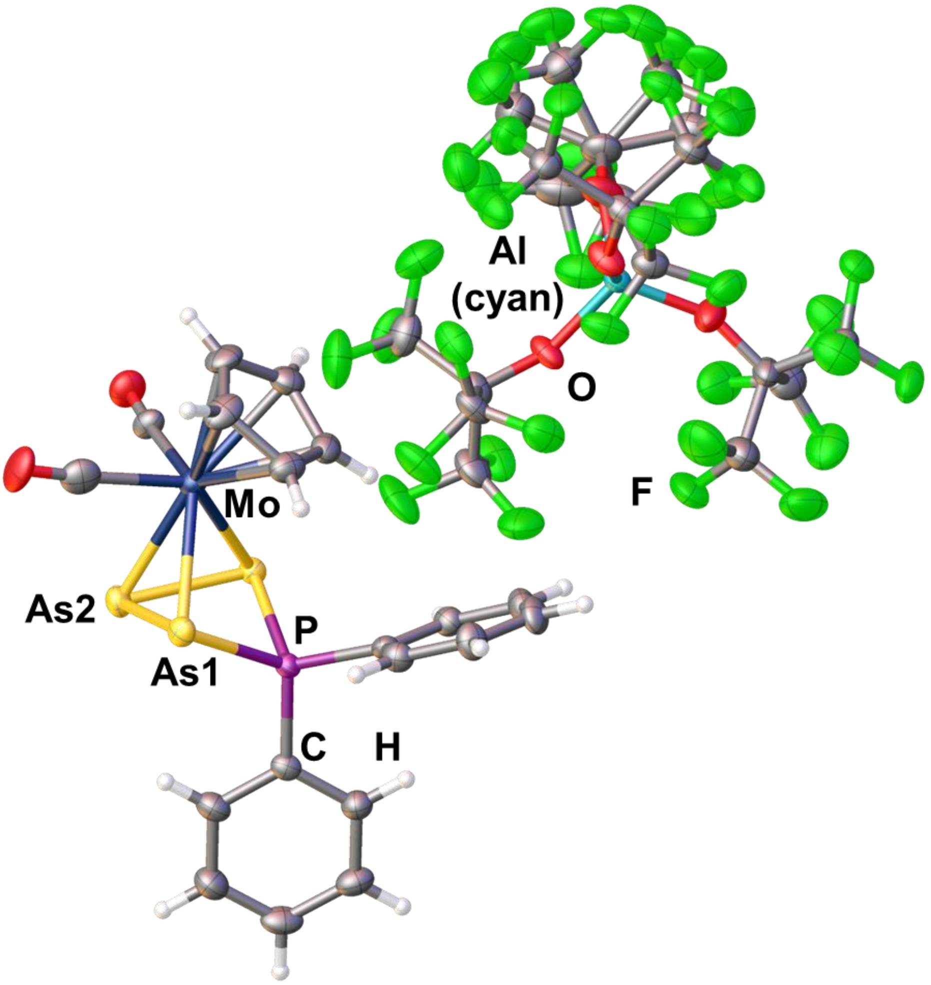

*Figure S 25: Solid state structure of **4**; Depicted is the asymmetric unit and ADPs (anisotropic displacement parameters) are drawn at 50 % probability.*

*[{CpMo(CO)$_2$}(η^3-As$_4$Cy$_2$)][TEF] (**5**)*

5 crystallizes as bright yellow sticks from concentrated CH_2Cl_2 solutions layered with *n*-hexane and storage at room temperature for seven days (Figure S 30). **5** crystallizes in the monoclinic space group *Pn* with two heavily disordered cations and anions in the asymmetric unit. Disorder was treated with adequate restraints.

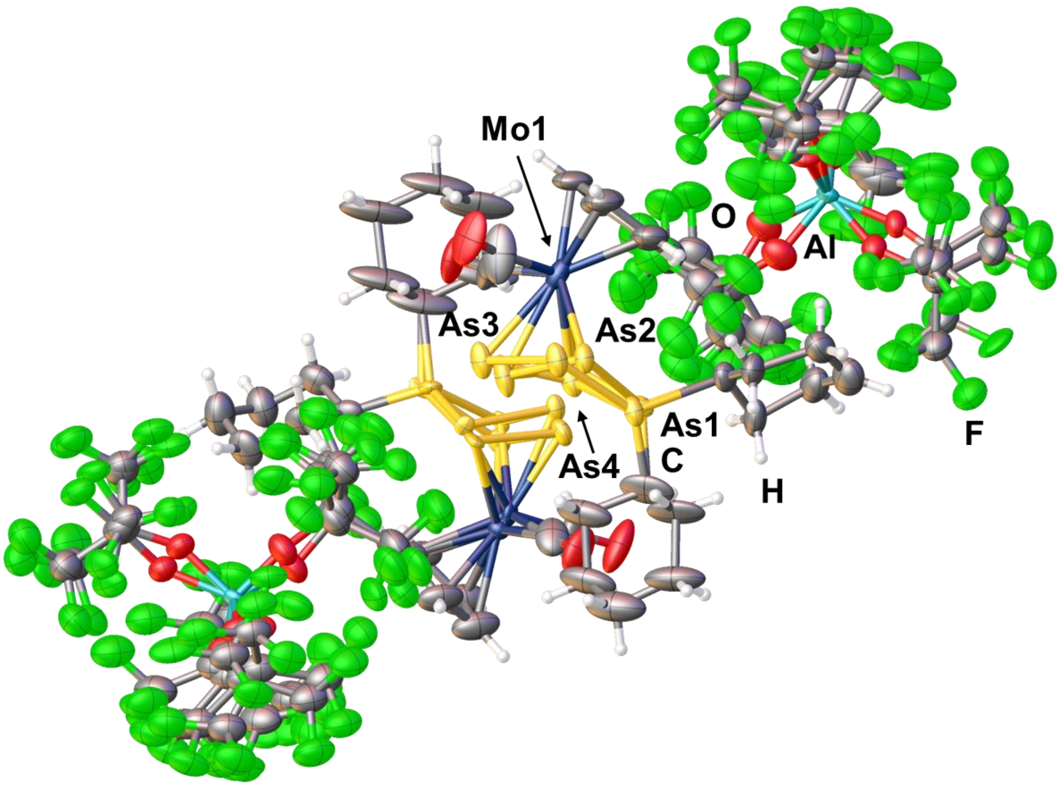

*Figure S 26: Solid state structure of **5**; Depicted is the asymmetric unit and ADPs (anisotropic displacement parameters) are drawn at 50 % probability.*

Quantum Crystallography

A Hirshfeld-Atom-Refinement (HAR) was performed on the single crystal X-ray diffraction dataset of **1** and compared to the HAR model of $\mathbf{A_{Ni}}$ which was previously extensively studied utilizing multipolar modeling and X-ray restrained wavefunction fitting.[14–16]

For the HAR procedure in NoSpherA2[17] within Olex2,[11] the geometry of the standard Independent-Atom model (IAM) was used as an input for the quantum chemical software ORCA.[18–22] A DFT calculation was conducted using the ωB97-ωB97X[23,24]/def2-TZVP[25] combination of the level of theory and basis sets. The criterion for convergence of the self-consistent-field (scf) procedure was set with the keyword "TightScf" and the grid accuracy to high (keyword "grid2"). An explicit solvation model using dichloromethane (cpcm = "CH2CL2")[26] was applied. The obtained wavefunction was then used to obtain non-spherical atomic scattering factors using the Hirshfeld-stockholder partitioning.[27] Then, geometry and anisotropic displacement parameters were adjusted in the standard least-squares procedure using the updated, non-spherical structure factors in olex2.refine. This procedure was repeated until convergence was achieved.

NoSpherA2 in Olex2 was then used to obtain the deformation density map, which describes the difference in Fourier maps between the IAM and HAR according to ρ(deform) = ρ(HAR) – ρ(IAM). The deformation density is an established indicator for shifts in electron density due to the system's chemical behavior.[28]

Due to disorder in the weakly coordinated anion $[TEF]^-$ a superposition of each of the geometries' resulting wavefunction was used, weighted according to their crystallographic site occupancy factors. For the analysis of the resulting wavefunction in the topological analysis, only the wavefunction associated with the main part (~0.63 in PART1 vs ~0.27 in PART2) was used. Since the disorder only occurs in the anion, we assume this is an appropriate approximation.

Additionally, multiwfn was employed to investigate the wavefunctions based on the experimental geometry. This was conducted by the means of Bader charges according to the Quantum Theory of Atoms in Molecules (QTAIM)[29] and the descriptors of the topology of the total electron density such as critical points in the electron density as well as the second derivative (Laplacian) of the total electron density, providing insights into the chemical fingerprints in positive (electron density depletion) and negative (electron density accumulation) areas.

Table 1 shows the resulting quality indicators of the HAR model of **1** and $\mathbf{A_{Ni}}$. There is a significant improvement in the agreement factors and residual density peaks after employing HAR. This indicates that the information gained by employing non-spherical, DFT-derived atomic form factors is in agreement with the measured intensities.

Figure S27 shows the residual electron density of the unit cell of **1** after HAR. The main residual electron density lies in the proximity of the arsenic atom, which is also the heaviest in the structure. Arsenic is known for showing strong residual density in crystal structures, partially due to absorption effects.

The residual electron density map can be improved around the As atom by employing a 3rd-order Gram-Charlier expansion on the anisotropic displacement parameters. While the resulting probability distribution function as provided by Olex2 is physically well-defined and no negative areas were found, Kuhs' rule[30] was unfulfilled. Here, a resolution of the diffraction data of 0.56 Å would have been required according to this rule, where 0.80 Å data was

obtained. Additionally, there was no significant improvement in quality indicators. Therefore, the classical harmonical anisotropic description was chosen.

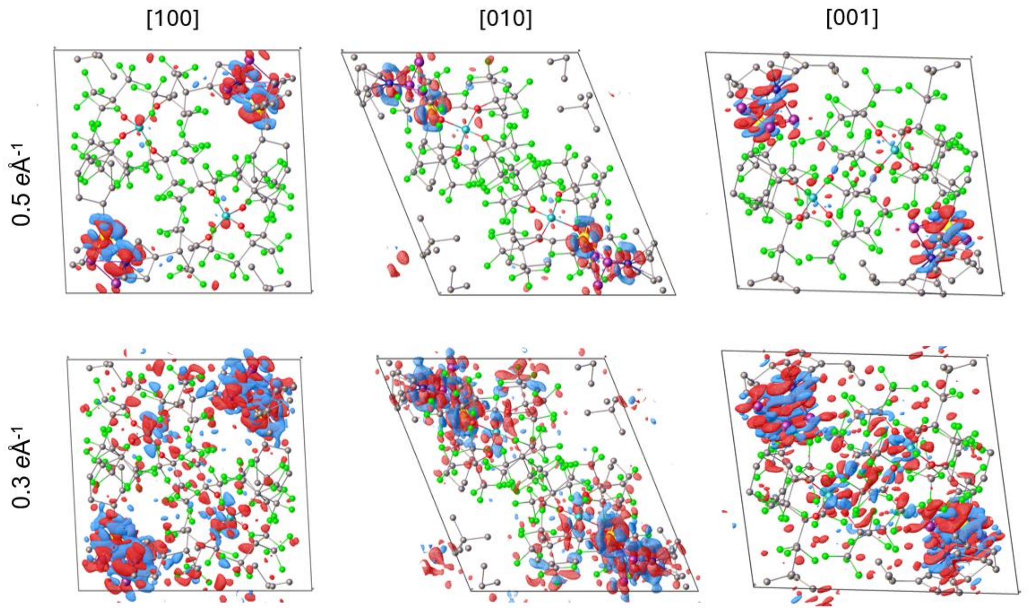

*Figure S 27: Residual electron density of the HAR models of **1** in the crystallographic a, b, and c direction at the 0.5 eÅ^{-1} (top) and 0.3 eÅ^{-1} (bottom) levels. Red indicates negative, blue positive residual density.*

Figure S28 compares the deformation density for different parts of **1** and $\mathbf{A_{Ni}}$.

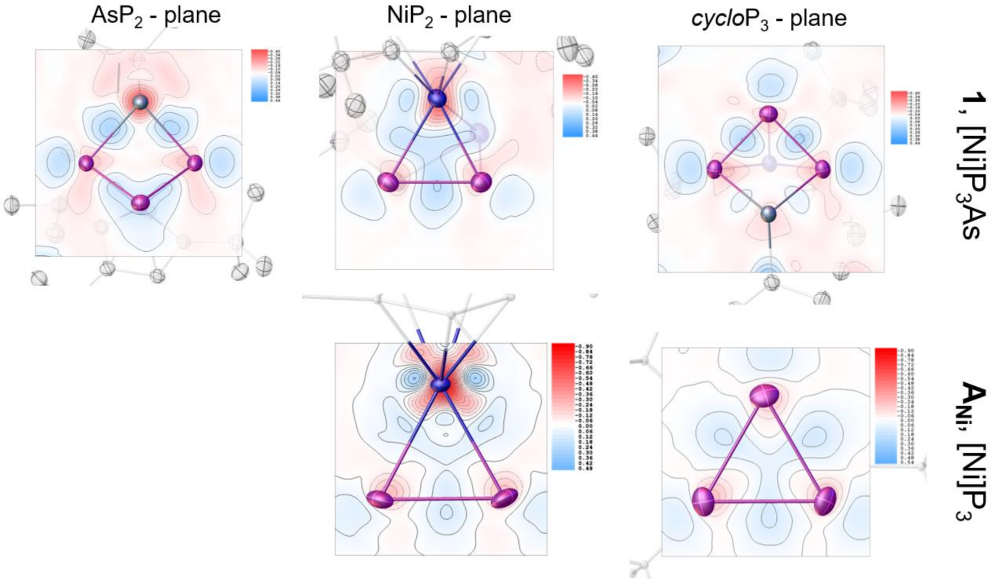

*Figure S 28: Contour maps of the dynamic deformation density of the HAR models of **1** in the crystallographic AsP2, NiP2, and cycloP3 plane at iso-levels indicated in the respective figure legends. Red areas indicate negative, blue positive deformation density, and therefore electron depletion or accumulation, respectively.*

The deformation density shows the effects of the P3 ring-opening analogous to the Laplacian plots (see Fig. 3 in the main text). There is significantly less deformation density in the opened P-P bond in **1** as compared to the remaining two bonds and all three P-P bonds in $\mathbf{A_{Ni}}$.

The coordination to the nickel center reveals a distortion in deformation density in **1** as compared to $\mathbf{A_{Ni}}$.

The P-As bond in **1** shows strong similarities to the P-P bonds in 1, with a more pronounced density depletion around the arsenic atom.

Bader Charges of **1**

All charges were obtained via multiwfn[31] on either the resulting HAR wavefunction (.gbw from the ORCA output) or the gas-phase optimized comparison structures for an arsenium and an arsonium representative (see below). Structures were optimized using the r2SCAN-3c composite method and basis set.[32] The charges were obtained from a single-point calculation on the final geometry employing the same settings as **1**. The integration in multiwfn was performed on the total electron density, with a high-quality grid (resolution of 0.06 bohr), and integrated according to the AIM approach (option 7).

1 (As)	Charge:	0.824951	Volume:	110.713 Bohr^3
2 (Ni)	Charge:	0.452027	Volume:	79.912 Bohr^3
3 (P)	Charge:	-0.068166	Volume:	196.215 Bohr^3
4 (P)	Charge:	-0.078104	Volume:	183.795 Bohr^3
5 (P)	Charge:	-0.010902	Volume:	192.576 Bohr^3
6 (Al)	Charge:	2.592095	Volume:	23.406 Bohr^3
7 (F)	Charge:	-0.660965	Volume:	98.005 Bohr^3
8 (F)	Charge:	-0.661665	Volume:	96.878 Bohr^3
9 (F)	Charge:	-0.662332	Volume:	100.243 Bohr^3
10 (F)	Charge:	-0.660546	Volume:	96.198 Bohr^3
11 (F)	Charge:	-0.657101	Volume:	97.753 Bohr^3
12 (F)	Charge:	-0.650210	Volume:	100.621 Bohr^3
13 (F)	Charge:	-0.658159	Volume:	99.433 Bohr^3
14 (F)	Charge:	-0.655365	Volume:	100.259 Bohr^3
15 (F)	Charge:	-0.649874	Volume:	99.409 Bohr^3
16 (F)	Charge:	-0.662675	Volume:	97.168 Bohr^3
17 (F)	Charge:	-0.662084	Volume:	98.338 Bohr^3
18 (F)	Charge:	-0.659887	Volume:	98.439 Bohr^3
19 (F)	Charge:	-0.653241	Volume:	100.029 Bohr^3
20 (F)	Charge:	-0.664193	Volume:	102.460 Bohr^3
21 (F)	Charge:	-0.660385	Volume:	99.042 Bohr^3
22 (F)	Charge:	-0.657953	Volume:	97.405 Bohr^3
23 (F)	Charge:	-0.655495	Volume:	97.741 Bohr^3
24 (O)	Charge:	-1.372054	Volume:	100.146 Bohr^3
25 (F)	Charge:	-0.655204	Volume:	98.207 Bohr^3
26 (F)	Charge:	-0.660932	Volume:	99.292 Bohr^3
27 (F)	Charge:	-0.654906	Volume:	98.674 Bohr^3
28 (O)	Charge:	-1.366312	Volume:	99.705 Bohr^3
29 (F)	Charge:	-0.656050	Volume:	98.528 Bohr^3
30 (O)	Charge:	-1.364359	Volume:	99.676 Bohr^3
31 (F)	Charge:	-0.659870	Volume:	100.191 Bohr^3
32 (O)	Charge:	-1.354711	Volume:	98.908 Bohr^3
33 (F)	Charge:	-0.657729	Volume:	97.874 Bohr^3
34 (F)	Charge:	-0.661950	Volume:	97.638 Bohr^3
35 (F)	Charge:	-0.659572	Volume:	97.904 Bohr^3
36 (F)	Charge:	-0.651096	Volume:	98.159 Bohr^3
37 (F)	Charge:	-0.643306	Volume:	99.790 Bohr^3
38 (F)	Charge:	-0.668617	Volume:	102.382 Bohr^3
39 (F)	Charge:	-0.647374	Volume:	100.739 Bohr^3
40 (F)	Charge:	-0.658803	Volume:	99.690 Bohr^3
41 (F)	Charge:	-0.658079	Volume:	98.633 Bohr^3
42 (F)	Charge:	-0.638014	Volume:	99.060 Bohr^3
43 (F)	Charge:	-0.658630	Volume:	96.903 Bohr^3
44 (F)	Charge:	-0.651073	Volume:	97.168 Bohr^3
45 (C)	Charge:	-0.149372	Volume:	73.359 Bohr^3
46 (H)	Charge:	0.109876	Volume:	41.605 Bohr^3
47 (C)	Charge:	-0.316003	Volume:	57.992 Bohr^3
48 (H)	Charge:	0.045939	Volume:	47.779 Bohr^3
49 (C)	Charge:	-0.100528	Volume:	54.324 Bohr^3
50 (F)	Charge:	-0.641279	Volume:	101.472 Bohr^3
51 (C)	Charge:	-0.111729	Volume:	56.866 Bohr^3
52 (C)	Charge:	-0.131724	Volume:	70.816 Bohr^3
53 (H)	Charge:	0.074912	Volume:	42.665 Bohr^3
54 (C)	Charge:	0.067395	Volume:	53.581 Bohr^3
55 (H)	Charge:	0.005995	Volume:	47.787 Bohr^3
56 (H)	Charge:	-0.016484	Volume:	44.452 Bohr^3
57 (C)	Charge:	0.104838	Volume:	42.770 Bohr^3
58 (C)	Charge:	0.099501	Volume:	42.239 Bohr^3
59 (C)	Charge:	-0.112153	Volume:	59.540 Bohr^3
60 (C)	Charge:	0.102383	Volume:	41.804 Bohr^3
61 (C)	Charge:	-0.336349	Volume:	58.789 Bohr^3
62 (H)	Charge:	0.057553	Volume:	48.612 Bohr^3
63 (C)	Charge:	0.771784	Volume:	32.239 Bohr^3
64 (C)	Charge:	0.766635	Volume:	32.516 Bohr^3
65 (C)	Charge:	0.016814	Volume:	62.599 Bohr^3

66 (H)	Charge:	-0.013898	Volume:	45.767 Bohr^3
67 (H)	Charge:	0.007159	Volume:	42.069 Bohr^3
68 (H)	Charge:	0.001655	Volume:	47.934 Bohr^3
69 (C)	Charge:	0.003979	Volume:	57.209 Bohr^3
70 (H)	Charge:	0.016135	Volume:	49.303 Bohr^3
71 (H)	Charge:	0.024764	Volume:	48.156 Bohr^3
72 (C)	Charge:	0.007293	Volume:	64.951 Bohr^3
73 (H)	Charge:	0.010244	Volume:	48.748 Bohr^3
74 (H)	Charge:	-0.006598	Volume:	48.468 Bohr^3
75 (H)	Charge:	0.010525	Volume:	50.255 Bohr^3
76 (C)	Charge:	0.033560	Volume:	57.994 Bohr^3
77 (H)	Charge:	0.007065	Volume:	50.434 Bohr^3
78 (H)	Charge:	-0.012166	Volume:	48.734 Bohr^3
79 (C)	Charge:	0.016060	Volume:	58.583 Bohr^3
80 (H)	Charge:	0.008017	Volume:	49.523 Bohr^3
81 (H)	Charge:	-0.001781	Volume:	51.044 Bohr^3
82 (C)	Charge:	1.862594	Volume:	19.824 Bohr^3
83 (C)	Charge:	0.068865	Volume:	53.698 Bohr^3
84 (H)	Charge:	-0.020432	Volume:	44.527 Bohr^3
85 (H)	Charge:	0.014145	Volume:	45.101 Bohr^3
86 (C)	Charge:	-0.006686	Volume:	65.438 Bohr^3
87 (H)	Charge:	0.013065	Volume:	49.385 Bohr^3
88 (H)	Charge:	0.007177	Volume:	46.491 Bohr^3
89 (H)	Charge:	0.006769	Volume:	48.627 Bohr^3
90 (C)	Charge:	1.872607	Volume:	19.770 Bohr^3
91 (C)	Charge:	1.863006	Volume:	19.847 Bohr^3
92 (C)	Charge:	1.870931	Volume:	19.647 Bohr^3
93 (C)	Charge:	0.010378	Volume:	64.127 Bohr^3
94 (H)	Charge:	0.002143	Volume:	47.932 Bohr^3
95 (H)	Charge:	0.002847	Volume:	49.563 Bohr^3
96 (H)	Charge:	0.006507	Volume:	50.779 Bohr^3
97 (C)	Charge:	0.772553	Volume:	32.178 Bohr^3
98 (C)	Charge:	1.881628	Volume:	19.641 Bohr^3
99 (C)	Charge:	0.016802	Volume:	62.901 Bohr^3
100 (H)	Charge:	0.001134	Volume:	39.968 Bohr^3
101 (H)	Charge:	0.014927	Volume:	46.174 Bohr^3
102 (H)	Charge:	-0.005581	Volume:	46.228 Bohr^3
103 (C)	Charge:	0.021754	Volume:	56.461 Bohr^3
104 (H)	Charge:	0.009531	Volume:	48.060 Bohr^3
105 (H)	Charge:	0.014960	Volume:	47.807 Bohr^3
106 (C)	Charge:	1.866868	Volume:	19.767 Bohr^3
107 (C)	Charge:	0.756519	Volume:	32.574 Bohr^3
108 (C)	Charge:	0.058726	Volume:	62.642 Bohr^3
109 (H)	Charge:	-0.003682	Volume:	48.121 Bohr^3
110 (H)	Charge:	-0.008048	Volume:	48.423 Bohr^3
111 (H)	Charge:	-0.011731	Volume:	43.019 Bohr^3
112 (C)	Charge:	0.053152	Volume:	56.759 Bohr^3
113 (H)	Charge:	0.006139	Volume:	49.312 Bohr^3
114 (H)	Charge:	-0.013564	Volume:	48.588 Bohr^3
115 (C)	Charge:	0.031843	Volume:	62.514 Bohr^3
116 (H)	Charge:	-0.011196	Volume:	42.066 Bohr^3
117 (H)	Charge:	-0.009111	Volume:	49.081 Bohr^3
118 (H)	Charge:	-0.001194	Volume:	47.866 Bohr^3
119 (C)	Charge:	1.873299	Volume:	19.812 Bohr^3
120 (C)	Charge:	1.877253	Volume:	19.584 Bohr^3
121 (C)	Charge:	-0.030734	Volume:	64.259 Bohr^3
122 (H)	Charge:	0.012451	Volume:	47.937 Bohr^3
123 (H)	Charge:	0.011852	Volume:	49.878 Bohr^3
124 (H)	Charge:	0.022630	Volume:	48.798 Bohr^3
125 (C)	Charge:	0.012707	Volume:	64.299 Bohr^3
126 (H)	Charge:	0.017148	Volume:	46.277 Bohr^3
127 (H)	Charge:	-0.018258	Volume:	44.379 Bohr^3
128 (H)	Charge:	0.003705	Volume:	48.496 Bohr^3
129 (C)	Charge:	0.003511	Volume:	58.918 Bohr^3
130 (H)	Charge:	-0.002945	Volume:	51.273 Bohr^3
131 (H)	Charge:	0.010594	Volume:	52.437 Bohr^3
132 (C)	Charge:	1.886350	Volume:	19.422 Bohr^3
133 (C)	Charge:	0.006774	Volume:	60.397 Bohr^3
134 (H)	Charge:	0.015471	Volume:	51.541 Bohr^3
135 (H)	Charge:	0.021521	Volume:	49.785 Bohr^3
136 (C)	Charge:	0.011165	Volume:	59.102 Bohr^3
137 (H)	Charge:	0.009552	Volume:	50.790 Bohr^3
138 (H)	Charge:	0.002607	Volume:	51.335 Bohr^3
139 (C)	Charge:	1.843656	Volume:	20.070 Bohr^3
140 (C)	Charge:	1.842400	Volume:	20.074 Bohr^3
141 (C)	Charge:	1.862931	Volume:	19.999 Bohr^3
142 (F)	Charge:	-0.653130	Volume:	97.568 Bohr^3

Bader Charges of $\mathbf{A_{Ni}}$

1 (Ni)	Charge:	0.421565	Volume:	81.508 Bohr^3
2 (P)	Charge:	-0.087215	Volume:	209.263 Bohr^3
3 (P)	Charge:	-0.095532	Volume:	207.892 Bohr^3
4 (P)	Charge:	-0.090501	Volume:	210.293 Bohr^3
5 (C)	Charge:	-0.124456	Volume:	60.289 Bohr^3
6 (C)	Charge:	-0.129320	Volume:	71.634 Bohr^3
7 (C)	Charge:	-0.120132	Volume:	70.872 Bohr^3
8 (C)	Charge:	-0.115655	Volume:	58.646 Bohr^3
9 (C)	Charge:	-0.108800	Volume:	56.530 Bohr^3
10 (C)	Charge:	0.114838	Volume:	41.685 Bohr^3
11 (C)	Charge:	0.108475	Volume:	42.435 Bohr^3
12 (C)	Charge:	0.063938	Volume:	61.890 Bohr^3
13 (C)	Charge:	0.108104	Volume:	42.603 Bohr^3
14 (C)	Charge:	0.059421	Volume:	63.357 Bohr^3
15 (C)	Charge:	0.064384	Volume:	62.992 Bohr^3
16 (C)	Charge:	0.058163	Volume:	64.871 Bohr^3
17 (C)	Charge:	0.065592	Volume:	61.833 Bohr^3
18 (C)	Charge:	0.062391	Volume:	64.310 Bohr^3
19 (C)	Charge:	0.065186	Volume:	63.352 Bohr^3
20 (C)	Charge:	0.071262	Volume:	63.433 Bohr^3
21 (C)	Charge:	0.058277	Volume:	61.321 Bohr^3
22 (H)	Charge:	0.050614	Volume:	42.307 Bohr^3
23 (H)	Charge:	0.050669	Volume:	42.408 Bohr^3
24 (H)	Charge:	-0.023597	Volume:	46.412 Bohr^3
25 (H)	Charge:	-0.019374	Volume:	50.393 Bohr^3
26 (H)	Charge:	-0.019375	Volume:	48.557 Bohr^3
27 (H)	Charge:	-0.023911	Volume:	47.947 Bohr^3
28 (H)	Charge:	-0.022001	Volume:	48.654 Bohr^3
29 (H)	Charge:	-0.015167	Volume:	49.970 Bohr^3
30 (H)	Charge:	-0.010910	Volume:	42.110 Bohr^3
31 (H)	Charge:	-0.026720	Volume:	46.811 Bohr^3
32 (H)	Charge:	-0.028467	Volume:	49.245 Bohr^3
33 (H)	Charge:	-0.016786	Volume:	50.827 Bohr^3
34 (H)	Charge:	-0.020745	Volume:	50.055 Bohr^3
35 (H)	Charge:	-0.017309	Volume:	49.935 Bohr^3
36 (H)	Charge:	-0.021853	Volume:	47.841 Bohr^3
37 (H)	Charge:	-0.022803	Volume:	41.488 Bohr^3
38 (H)	Charge:	-0.017252	Volume:	48.883 Bohr^3
39 (H)	Charge:	-0.018345	Volume:	47.186 Bohr^3
40 (H)	Charge:	-0.021213	Volume:	49.623 Bohr^3
41 (H)	Charge:	-0.017597	Volume:	49.740 Bohr^3
42 (H)	Charge:	-0.026235	Volume:	47.984 Bohr^3
43 (H)	Charge:	-0.019142	Volume:	49.185 Bohr^3
44 (H)	Charge:	-0.018784	Volume:	49.693 Bohr^3
45 (H)	Charge:	-0.024231	Volume:	50.403 Bohr^3
46 (H)	Charge:	-0.023635	Volume:	42.635 Bohr^3
47 (H)	Charge:	-0.019568	Volume:	49.141 Bohr^3
48 (H)	Charge:	-0.016464	Volume:	49.330 Bohr^3
49 (H)	Charge:	-0.020703	Volume:	48.581 Bohr^3
50 (H)	Charge:	-0.019082	Volume:	48.645 Bohr^3

DFT Optimized Geometries for the Comparison of Bader Charges

Cy_2As^+ - Model Arsenium

As	16.36073960156414	11.64523369037261	10.94765003341947
C	16.72001099032580	11.44596048789974	9.04715843738583
H	17.41266713575929	10.59708784123556	8.94504432622984
C	14.36593952044906	13.41158788151171	12.19392888739312
H	14.82264231801040	14.38270912005719	11.95401509126833
H	14.87675065322103	13.03151923246018	13.09613822764531
C	14.59080544761511	12.44436418474380	11.02969146086593
H	14.25554333011572	12.85390681091235	10.06869815720945
C	13.91867729704110	11.04950898584022	11.27195833963328
H	13.99885966950201	10.39697878390877	10.38924004146129
H	14.37284325157943	10.52038157168105	12.13478787603724
C	12.20088238914567	12.22806352029344	12.76671504723010
H	11.12104816677412	12.36290117357752	12.93506899823438
H	12.61310952549429	11.79032044302469	13.69220855796230
C	12.86902382199741	13.58099902194476	12.49322346679507
H	12.74273051653514	14.25330722185494	13.35497380341521
H	12.37664452810430	14.06743009474893	11.63380211022941
C	17.56244764045098	12.77275991354234	9.02667670477306
H	18.36435974035984	12.79492919842342	9.78763320479910
H	16.91288792448226	13.65026342463311	9.18151470284857
C	15.65511757978648	11.42927507010304	7.96000563879863
H	14.93648792736473	12.25059985805124	8.11263724949992
H	15.08948692228126	10.48706324093973	8.00559694387090
C	12.40964993123566	11.25855185093070	11.60564646068624
H	11.91985883670609	11.64250488599967	10.69644141381898
H	11.98168971302032	10.26963453017170	11.82664097685019
C	18.22873666558048	12.89202390560504	7.62201342236164
H	18.79342986800127	13.83611760915064	7.60583814436681
H	18.94917799875887	12.06716952077430	7.50827982303596
C	17.18092471168874	12.85125324550680	6.50963198339774
H	16.53474327355813	13.74262678621264	6.58381965300899
H	17.68930243579436	12.90946212893947	5.53428915494977
C	16.31758598516084	11.58464999254470	6.57831693349191
H	16.94438820510179	10.69979690248778	6.37629832056946
H	15.53960647743348	11.60955786991576	5.80091640645626

Cy_2As^+ - Bader Charges

1 (As)	Charge:	1.131567	Volume:	173.565 Bohr^3
2 (C)	Charge:	-0.353564	Volume:	61.998 Bohr^3
3 (H)	Charge:	0.047420	Volume:	45.455 Bohr^3
4 (C)	Charge:	0.077015	Volume:	56.971 Bohr^3
5 (H)	Charge:	0.002713	Volume:	48.428 Bohr^3
6 (H)	Charge:	-0.011335	Volume:	49.553 Bohr^3
7 (C)	Charge:	-0.352542	Volume:	61.672 Bohr^3
8 (H)	Charge:	0.049693	Volume:	44.370 Bohr^3
9 (C)	Charge:	-0.031581	Volume:	57.370 Bohr^3
10 (H)	Charge:	0.019102	Volume:	46.802 Bohr^3
11 (H)	Charge:	0.005981	Volume:	47.393 Bohr^3
12 (C)	Charge:	0.074647	Volume:	57.279 Bohr^3
13 (H)	Charge:	-0.009511	Volume:	49.059 Bohr^3
14 (H)	Charge:	-0.022144	Volume:	50.137 Bohr^3
15 (C)	Charge:	0.073063	Volume:	57.465 Bohr^3
16 (H)	Charge:	-0.009245	Volume:	49.007 Bohr^3
17 (H)	Charge:	-0.020759	Volume:	50.024 Bohr^3
18 (C)	Charge:	-0.035307	Volume:	57.411 Bohr^3
19 (H)	Charge:	0.016741	Volume:	46.434 Bohr^3
20 (H)	Charge:	0.014652	Volume:	47.385 Bohr^3
21 (C)	Charge:	0.082288	Volume:	56.401 Bohr^3
22 (H)	Charge:	-0.008803	Volume:	46.891 Bohr^3
23 (H)	Charge:	0.004117	Volume:	48.719 Bohr^3
24 (C)	Charge:	0.084751	Volume:	57.448 Bohr^3
25 (H)	Charge:	-0.006761	Volume:	49.031 Bohr^3
26 (H)	Charge:	0.002819	Volume:	48.233 Bohr^3
27 (C)	Charge:	0.085495	Volume:	57.443 Bohr^3
28 (H)	Charge:	0.004317	Volume:	48.186 Bohr^3
29 (H)	Charge:	-0.004227	Volume:	48.769 Bohr^3
30 (C)	Charge:	0.072675	Volume:	57.257 Bohr^3
31 (H)	Charge:	-0.022144	Volume:	49.984 Bohr^3
32 (H)	Charge:	-0.008132	Volume:	49.009 Bohr^3
33 (C)	Charge:	0.074427	Volume:	57.402 Bohr^3
34 (H)	Charge:	-0.019680	Volume:	49.771 Bohr^3
35 (H)	Charge:	-0.007749	Volume:	48.932 Bohr^3

Cy_4As^+ - Model Arsonium

As	-1.23457040789991	2.50973286869609	-1.04855289082012
C	-2.97286572364964	3.15034498984557	-0.37500873059265
C	-0.00515363354178	3.89756977148150	-0.36717148974932
C	-0.73641015151014	0.78624905970575	-0.22541904398466
C	0.43756604950539	0.10972884185308	-0.94901825961900
C	0.86870725019711	-1.14233128375228	-0.17612304972128
C	-0.29847005566103	-2.11616766406722	-0.01030569496716
C	-1.48495673127039	-1.43473252885390	0.67149431458237
C	-1.92397809149888	-0.17626027242463	-0.08799489879367
C	-4.21147434689944	2.53952720527813	-1.03654635750665
C	-5.46764253858296	3.32977025416801	-0.60834605157844
C	-5.38257400085922	3.83488524997775	0.84729715755452
C	-4.46666456726279	2.93582515835284	1.67415146083746
C	-3.01759735915512	3.05863635171952	1.17285459720536
C	-0.20929371223854	5.23294570021235	-1.09569226836088
C	0.64887152741762	6.32323609202345	-0.44231462849243
C	2.12342212821640	5.92094782797042	-0.41852072340248
C	2.30997370660323	4.59166004817806	0.31256042165706
C	1.46741840655344	3.47755804278813	-0.32200779471950
H	-2.94317175129694	4.20834554846817	-0.66731916144296
H	-0.35645599841734	4.01046041889182	0.66931674242384
H	-0.40415901974657	1.08828226415652	0.77895722287173
H	0.12619945301479	-0.18844486290495	-1.95645430594155
H	1.28571516166277	0.79084396465532	-1.06166208086267
H	1.69940108486033	-1.62339780835336	-0.70415807650443
H	1.24424792703668	-0.84497701621062	0.81249051057506
H	-0.60858562529046	-2.47988140275372	-0.99973367936760
H	0.01777586759759	-2.99154548732514	0.56779361610426
H	-2.33350887645942	-2.12353165434536	0.74795353236764
H	-1.20574435159808	-1.15290654818661	1.69593884637116
H	-2.28768947454051	-0.45954309388867	-1.08364836682152
H	-2.75715774894193	0.29669473601296	0.44020207103835
H	-4.12698647210830	2.57455804337771	-2.12573471343978
H	-4.31131650350983	1.48632998831046	-0.75565300755071
H	-5.60891531067831	4.17867234029180	-1.28607279687172
H	-6.33994325683147	2.67874359646106	-0.73024181822083
H	-4.98069107202500	4.85616050517267	0.86813468975970
H	-6.38339738328364	3.88461993915009	1.28715907885041
H	-4.50177712912119	3.20223707310132	2.73529119708146
H	-4.80478144624743	1.89486978972308	1.59700600615208
H	-2.56606816655912	3.96464988016374	1.59106695446912
H	-2.43167424162064	2.21391955319368	1.55110664972435
H	0.08294624559499	5.13585268651121	-2.14804545640056
H	-1.26278454768651	5.53176541517597	-1.07969272982847
H	0.51324947442666	7.26401852835068	-0.98701478086653
H	0.29790164229291	6.49057654004419	0.58501527855601
H	2.48814622422054	5.82273837808618	-1.45053500367400
H	2.72212132012791	6.70338056209001	0.06081190641301
H	3.36389622834459	4.29248808529440	0.30828158607559
H	2.01348602310317	4.70967534893027	1.36383335690557
H	1.83746333212723	3.27580972368777	-1.33463202353093
H	1.58355023719461	2.55866638671317	0.26141342793513
C	-1.28678440499038	2.44806904245730	-3.02056002489043
C	0.09638218459299	2.57715198903760	-3.67364067055215
C	-0.05771441117426	2.65514836983283	-5.19822284079582
H	0.70975030053671	1.70566045501263	-3.41394198825456
H	0.62457680521816	3.46454948717070	-3.31636063760371
C	-0.81633129660054	1.44752926095536	-5.74945893114657
H	0.93477656681264	2.72792470983208	-5.65656442634523
H	-0.59851672193190	3.57617784626302	-5.45546506964428
C	-2.17474854863132	1.29596428788009	-5.06422988271287
H	-0.22363963232159	0.53850325173274	-5.57721025116518
H	-0.94604637353527	1.54541603330971	-6.83297311354646
C	-2.01350746162437	1.19818258318692	-3.54265075227724
H	-2.69317705950887	0.40312823837246	-5.43077370039215
H	-2.80920476393205	2.15989013992204	-5.30445913587597
H	-1.42050759823855	0.30645416538454	-3.30850364631973
H	-2.98922456073182	1.06808575006322	-3.06722105083416
H	-1.88392261804496	3.33954725438824	-3.26613461952213

Cy_4As^+ - Bader Charges

1 (As)	Charge:	1.282009	Volume:	92.524 Bohr^3
2 (C)	Charge:	-0.284552	Volume:	56.782 Bohr^3
3 (C)	Charge:	-0.279959	Volume:	56.567 Bohr^3
4 (C)	Charge:	-0.283477	Volume:	56.204 Bohr^3
5 (C)	Charge:	0.086541	Volume:	54.109 Bohr^3
6 (C)	Charge:	0.086072	Volume:	56.489 Bohr^3
7 (C)	Charge:	0.087509	Volume:	56.543 Bohr^3
8 (C)	Charge:	0.085865	Volume:	56.462 Bohr^3
9 (C)	Charge:	0.087033	Volume:	54.488 Bohr^3
10 (C)	Charge:	0.092436	Volume:	54.324 Bohr^3
11 (C)	Charge:	0.082046	Volume:	57.635 Bohr^3
12 (C)	Charge:	0.082495	Volume:	56.857 Bohr^3
13 (C)	Charge:	0.090386	Volume:	56.213 Bohr^3
14 (C)	Charge:	0.078423	Volume:	55.105 Bohr^3
15 (C)	Charge:	0.083872	Volume:	54.971 Bohr^3
16 (C)	Charge:	0.088069	Volume:	56.452 Bohr^3
17 (C)	Charge:	0.086732	Volume:	56.549 Bohr^3
18 (C)	Charge:	0.086377	Volume:	56.432 Bohr^3
19 (C)	Charge:	0.088687	Volume:	54.246 Bohr^3
20 (H)	Charge:	0.016269	Volume:	43.552 Bohr^3
21 (H)	Charge:	0.017779	Volume:	44.682 Bohr^3
22 (H)	Charge:	0.020360	Volume:	45.326 Bohr^3
23 (H)	Charge:	-0.021556	Volume:	44.684 Bohr^3
24 (H)	Charge:	-0.015795	Volume:	45.281 Bohr^3
25 (H)	Charge:	-0.020435	Volume:	48.972 Bohr^3
26 (H)	Charge:	-0.030326	Volume:	49.563 Bohr^3
27 (H)	Charge:	-0.034687	Volume:	49.789 Bohr^3
28 (H)	Charge:	-0.025864	Volume:	49.271 Bohr^3
29 (H)	Charge:	-0.020970	Volume:	48.986 Bohr^3
30 (H)	Charge:	-0.029837	Volume:	49.482 Bohr^3
31 (H)	Charge:	-0.018255	Volume:	47.382 Bohr^3
32 (H)	Charge:	-0.017678	Volume:	44.142 Bohr^3
33 (H)	Charge:	-0.017485	Volume:	44.749 Bohr^3
34 (H)	Charge:	-0.021129	Volume:	46.184 Bohr^3
35 (H)	Charge:	-0.027692	Volume:	49.227 Bohr^3
36 (H)	Charge:	-0.023698	Volume:	48.917 Bohr^3
37 (H)	Charge:	-0.035979	Volume:	49.235 Bohr^3
38 (H)	Charge:	-0.023322	Volume:	49.042 Bohr^3
39 (H)	Charge:	-0.019589	Volume:	48.829 Bohr^3
40 (H)	Charge:	-0.030805	Volume:	48.795 Bohr^3
41 (H)	Charge:	-0.015478	Volume:	48.038 Bohr^3
42 (H)	Charge:	-0.023457	Volume:	44.861 Bohr^3
43 (H)	Charge:	-0.021014	Volume:	45.899 Bohr^3
44 (H)	Charge:	-0.019250	Volume:	47.081 Bohr^3
45 (H)	Charge:	-0.021358	Volume:	48.991 Bohr^3
46 (H)	Charge:	-0.030823	Volume:	49.489 Bohr^3
47 (H)	Charge:	-0.034935	Volume:	49.867 Bohr^3
48 (H)	Charge:	-0.025310	Volume:	49.289 Bohr^3
49 (H)	Charge:	-0.021817	Volume:	49.024 Bohr^3
50 (H)	Charge:	-0.030972	Volume:	49.493 Bohr^3
51 (H)	Charge:	-0.021156	Volume:	47.191 Bohr^3
52 (H)	Charge:	-0.017160	Volume:	45.597 Bohr^3
53 (C)	Charge:	-0.283159	Volume:	56.863 Bohr^3
54 (C)	Charge:	0.088541	Volume:	54.637 Bohr^3
55 (C)	Charge:	0.085684	Volume:	56.473 Bohr^3
56 (H)	Charge:	-0.017805	Volume:	46.973 Bohr^3
57 (H)	Charge:	-0.019310	Volume:	44.040 Bohr^3
58 (C)	Charge:	0.086321	Volume:	56.552 Bohr^3
59 (H)	Charge:	-0.020518	Volume:	48.956 Bohr^3
60 (H)	Charge:	-0.030299	Volume:	49.483 Bohr^3
61 (C)	Charge:	0.086205	Volume:	56.526 Bohr^3
62 (H)	Charge:	-0.033998	Volume:	49.731 Bohr^3
63 (H)	Charge:	-0.025183	Volume:	49.280 Bohr^3
64 (C)	Charge:	0.086103	Volume:	54.150 Bohr^3
65 (H)	Charge:	-0.020592	Volume:	48.946 Bohr^3
66 (H)	Charge:	-0.029987	Volume:	49.524 Bohr^3
67 (H)	Charge:	-0.020873	Volume:	44.765 Bohr^3
68 (H)	Charge:	-0.016595	Volume:	44.968 Bohr^3
69 (H)	Charge:	0.022328	Volume:	46.322 Bohr^3

Computational Details

General Considerations

Quantum chemical calculations regarding the reaction mechanism of arsenium ion insertion were performed using ORCA version 5.0.3 developed by Neese *et al.*[18–22] Images of calculated structures were generated with ChemCraft.[33] Molecular geometries were optimized at the ωB97X-D4[23,24,34,35]/def2-TZVP[25] level of theory including implicit solvent correction (CPCM = CH_2Cl_2).[36] For all structures, subsequent analytical frequency analysis was carried out in order to determine the nature of stationary points on the potential energy surface. Approximate transition states were found using the *Nudged Elastic Band* method (NEB-TS) as implemented in ORCA followed by a geometry optimization and frequency analysis of the saddle point.[37]

Optimized Geometries

A'$_{Ni}$(Cp)

ωB97X-D4/def2-TZVP (CPCM (CH_2Cl_2)): Energies/H = -2726.12525318, Free Energies/H = -2726.16991588, ZPVE/ kcal/mol = 62.83

Ni	17.14628453905374	9.81820197853161	10.63673542819669
P	16.25244500221727	10.68130373942759	8.80468789864628
P	17.09059442672657	11.99758124839073	10.24429399837057
P	15.26897093114986	10.99424045847329	10.66038609425778
C	17.03359179571195	8.18400376012865	11.92154915300129
H	16.15652253263196	7.88343706322527	12.47550914857394
C	17.38067550583409	7.75437529490025	10.61011754815998
C	19.01162101540940	9.21317878342301	11.33601522376314
C	18.03713713675965	9.09175626814628	12.36995863108766
C	18.60614776369770	8.39120196073212	10.24812709062708
H	19.13082390193280	8.27510812522877	9.31140507191600
H	18.06020245447726	9.59215670203415	13.32675702672801
H	19.89710558093257	9.83062274498739	11.36953503511981
H	16.81578696146490	7.06843604037077	9.99643330755156

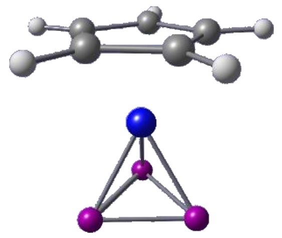

A'$_{Mo}$

ωB97X-D4/def2-TZVP (CPCM (CH_2Cl_2)): Energies/H = -1512.94501414, Free Energies/H = -1512.99743036, ZPVE/ kcal/mol = 76.66

Mo	-20.48807751357583	14.95626447984590	-8.78204592449987
P	-22.09091936593689	13.15892848473975	-9.40567712913801
P	-22.66495317622288	14.27344440566993	-7.68789751750273
P	-21.07028103610743	12.87828402254964	-7.54545477355726
H	-19.71935798123191	15.85172139224041	-11.54978400920761
C	-19.26955186002987	15.40967725303267	-10.67322788700639
C	-18.69688264047958	16.11941816198987	-9.57457518951763
H	-18.61834661668104	17.19147362070209	-9.47849559921482
H	-17.69740358727060	15.36096877729825	-7.71958175534337
C	-18.20041682390635	15.15017271412037	-8.65187254652165
C	-18.45876248435635	13.85974468486599	-9.18119671246200
H	-18.18952101566192	12.92093759688474	-8.72234025917633
C	-19.12022161247499	14.01972263867992	-10.42904492837931
H	-19.44031701054872	13.22186492571013	-11.08126457400925
C	-20.48463700747473	15.95751749318616	-7.05654927060363
O	-20.44446244041413	16.53929971664159	-6.07433213396703
C	-21.80416357258434	16.27776120865816	-9.50132264599833
O	-22.53315761104153	17.03958784918365	-9.93926269389454

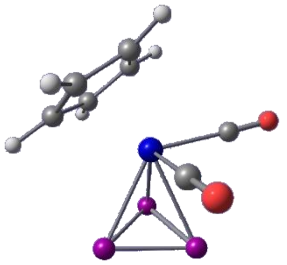

[$Me_2As]^+$

ωB97X-D4/def2-TZVP (CPCM (CH_2Cl_2)): Energies/H = -2315.41364017, Free Energies/H = -2315.44762742, ZPVE/ kcal/mol = 48.80

As	17.88872267537218	14.52654285782929	9.97749268629230
C	16.45730525190676	15.23037202358802	11.01288413731929
H	15.90178281189096	16.00168509287109	10.47838567679683
H	15.79612984671517	14.35676540615925	11.14363191023864
H	16.80401584133461	15.55406857072179	11.99276732703793
C	17.14153066277651	14.72875682760563	8.24142477861475
H	17.65222830761024	14.09740833658243	7.51709130409901
H	16.05990588455852	14.58575611945601	8.24704821136570
H	17.34542478083491	15.78619235118640	8.00382482923547

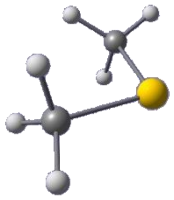

1'$_{Add}$(Cp,Me)

ωB97X-D4/def2-TZVP (CPCM (CH_2Cl_2)): Energies/H = -5041.59564426, Free Energies/H = -5041.65395759, ZPVE/ kcal/mol = 114.84

As	17.87869543435693	14.24506096987240	9.81098306989330
Ni	17.35516813168932	9.98297223914577	10.45530836723176
P	17.01216709179132	10.62758450304286	8.32387739184954
P	17.21955858891113	12.02203209234939	9.88404879505121
P	15.42987526718323	10.92582660416304	9.72365219571205
C	16.88331565928379	8.60017607570656	11.93535376745302
H	15.91890382482100	8.49770899859554	12.40968390009255
C	17.29819995288018	7.95239942894831	10.72889583936666
C	19.03070693463608	9.28534121779565	11.43314056214594
C	17.94020312902869	9.43915536993000	12.35971393601632
C	18.64182649373998	8.34851368847477	10.44529078988308
H	19.23499174454706	8.02394542686594	9.60361169963446
H	17.93464046232863	10.08548055483882	13.22471422082360
H	19.98004916883700	9.79735580561652	11.48671929355632
H	16.70371746748576	7.26729376007723	10.14277992206968
C	16.67406926980109	14.82332560049731	11.23305147132379
H	16.73199142141028	15.91407816278229	11.25420065810765
H	15.64845643430321	14.51212532759506	11.03872168144064
H	17.02146476143255	14.43078607781104	12.18860812733726
C	16.74464984766583	14.64367209872040	8.27278868293236
H	17.15691870378310	14.16517523902144	7.38489649009718
H	15.71635140169138	14.32484751948958	8.43971473399942
H	16.77903441939129	15.72829499265927	8.14630592098148

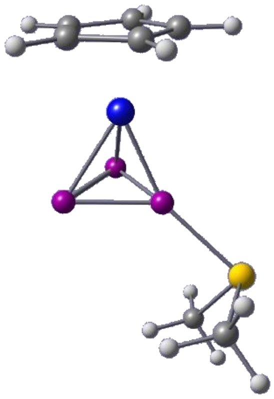

2’$_{Add}$(Me)

ωB97X-D4/def2-TZVP (CPCM (CH_2Cl_2)): Energies/H = -3828.42853607, Free Energies/H = -3828.49490135, ZPVE/ kcal/mol = 128.71

Mo	-19.89675190881790	12.05250680596830	-6.49374942097835
As	-21.95805615706086	7.97262366917640	-7.10565169336956
P	-22.34825559989154	11.50488861964728	-6.16968440496112
P	-21.13141208848098	10.12916341985233	-7.23446702353993
P	-21.79373194101877	11.89650484666449	-8.18934708888055
H	-17.58028421942593	13.58479851840736	-5.40236360644774
C	-17.85513915531485	13.00758969117358	-6.27240705820234
C	-17.65532045822416	11.60712449758093	-6.45159611867445
H	-17.21470548281712	10.93064640177245	-5.73423873364880
H	-18.07610254449896	10.27584314248067	-8.19277550524969
C	-18.10147310503914	11.26328259828504	-7.75700259858595
C	-18.58387209988881	12.43850909432696	-8.38786504792504
H	-18.97908934697281	12.50378273634704	-9.38983005144055
C	-18.44161332341863	13.51629018337601	-7.47414008525549
H	-18.69870974148269	14.54701523610334	-7.66902075677054
C	-19.85929317813911	11.37698041653850	-4.58859268126354
O	-19.79846672370020	10.99354733511543	-3.52313898448029
C	-20.69146126722928	13.76479811306614	-5.77706713435669
O	-21.12032723402906	14.73918809257466	-5.38432773302682
C	-21.12797478832540	7.34181068553825	-8.75718875249134
H	-21.36959735311484	7.99166959764731	-9.59789780587768
H	-20.04808032319017	7.28009339715927	-8.62141936844976
H	-21.52090944992566	6.33965995802645	-8.94264285112349
C	-23.71635845162945	8.42939904064219	-7.81703482791166
H	-24.28616087845379	8.96806580986427	-7.06033654862653
H	-23.63500672688526	9.02312611190141	-8.72738134989718
H	-24.21683087202290	7.48415214176290	-8.03965549456420

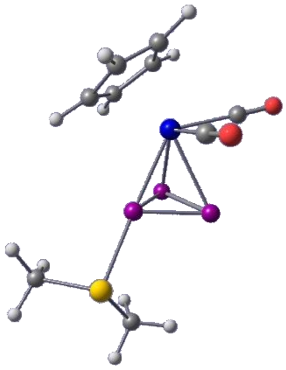

1’$_{TS}$(Cp,Me)

ωB97X-D4/def2-TZVP (CPCM (CH_2Cl_2)): Energies/H = -5041.58651608, Free Energies/H = -5041.64038565, ZPVE/ kcal/mol = 113.55

As	0.08516248714816	2.31112027579526	-1.05173968471990
Ni	-0.29636007510684	-0.76993726354946	1.09984447653285
P	-0.93555072053876	0.17074894525043	-0.75427877679734
P	-0.12126234007283	1.37069791391265	1.22426351346370
P	-2.07599341744694	0.59678062148290	1.01348692660439
C	-0.65833332515733	-2.34272436423857	2.39225274277932
H	-1.55261176671914	-2.45942630095721	2.98631568465699
C	-0.45235788160483	-2.85091972296941	1.08231310973271
C	1.45174618882922	-1.69956052228197	1.71950495221775
C	0.52230583534105	-1.63465741831964	2.78989098405337
C	0.84278579837234	-2.43627593791123	0.65630130692630
H	1.28992347845517	-2.64486758833780	-0.30445240959238
H	0.67141502787189	-1.13479371257202	3.73545886868639
H	2.43514062741364	-1.25409227683084	1.70051001863497
H	-1.16230781463830	-3.41904385677402	0.50012750231653
C	-1.43414128773345	2.89252956865187	-2.11803431278487
H	-1.63468535806003	2.16235524969976	-2.90245278640448
H	-2.29648518289521	2.99608099258247	-1.46004800167979
H	-1.17712410313547	3.86221005613123	-2.54507347866174
C	1.43866878170061	1.65713524865291	-2.27977277115431
H	2.14896778481682	1.05554051394188	-1.71287249593718
H	0.96308725995185	1.05509477374627	-3.05482607135870
H	1.94801000320833	2.51600480489454	-2.71671929751458

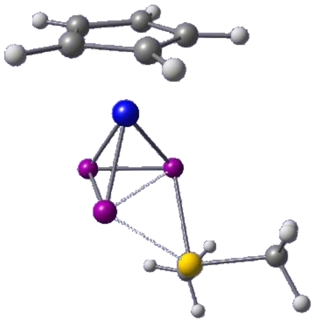

2’$_{TS}$(Me)

ωB97X-D4/def2-TZVP (CPCM (CH_2Cl_2)): Energies/H = -3828.40958119, Free Energies/H = -3828.47407235, ZPVE/ kcal/mol = 127.99

Mo	0.55999326450604	0.89513425769504	1.03392070614229
As	-0.78412979129374	-2.37819621931284	-0.61494657225116
P	0.69384897514026	-1.51053371085093	1.21014947942994
P	-1.49551866250709	-0.38096894943262	0.45844864322743
P	-0.79671522476000	-0.68197764172553	2.46830153081013
H	2.34560634464287	3.30817854326717	1.02286443159782
C	1.95953101184990	2.56322215244473	0.34319648592322
C	2.57305872800042	1.31110619233387	0.02292042898864
H	3.50194552665964	0.93596543255749	0.42610208152939
H	1.96003530510857	-0.29485046879778	-1.40054971485749
C	1.76074178373404	0.66226069614622	-0.94190958247593
C	0.65013025802661	1.50846810880876	-1.22582800047017
H	-0.14425520627159	1.30587670670245	-1.92757869297882
C	0.77728058748711	2.67764948877894	-0.44089671553881
H	0.09122583271255	3.51222623733346	-0.43496002963816
C	1.63044402024373	0.90600360635104	2.74849310207336
O	2.26160706271340	0.91028371621700	3.69149161322163
C	-0.77616866152221	2.13582123019605	1.91012275396252
O	-1.53046109080049	2.85159818845974	2.36341961076719
C	-2.39118289773343	-3.34748065779298	-0.11716723680222
H	-2.43996380943790	-4.26245268409432	-0.70807029203873
H	-2.32568133539115	-3.59688265129109	0.94190678944457
H	-3.26708205945767	-2.72358296903273	-0.30272128452117
C	-1.15563028498553	-1.66414861626400	-2.38793450577664
H	-0.32689600264730	-1.03583178620964	-2.70937685360060
H	-1.24612098311266	-2.51437980587802	-3.06484759524388
H	-2.08564269090440	-1.09250839660947	-2.36455058092439

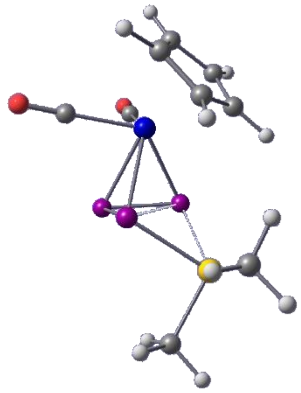

1’(Cp,Me)

ωB97X-D4/def2-TZVP (CPCM (CH_2Cl_2)): Energies/H = -5041.62745399, Free Energies/H = -5041.68357737, ZPVE/ kcal/mol = 114.95

As	17.14846697539093	12.24261455842650	8.32531391533384
Ni	17.01921455551242	10.08557324644072	10.38826844520170
P	15.81937327474085	10.41699597803342	8.52443770485543
P	17.05868129849382	12.31863040977612	10.59049507179934
P	15.17343129669728	11.30758969795579	10.37123457369415
C	17.07270653928904	8.45013506572340	11.74539102741123
H	16.26866139787470	8.14363261602949	12.39744041916006
C	17.26285369258108	8.03608939875894	10.40218071356017
C	18.93977284134245	9.53030129500311	10.89447142998870
C	18.06816615557685	9.41088052598965	12.03910314789938
C	18.44602737597684	8.67785532534425	9.88729242218723
H	18.85942988464212	8.54214397927468	8.89913958226719
H	18.17594823159760	9.95403331491670	12.96649469361894
H	19.80514322175232	10.17247687310915	10.82637060512655
H	16.64329555199189	7.33789452062756	9.85863632961424
C	16.31756088450990	13.72525295132342	7.43471263357661
H	16.21944159241363	13.47617560695223	6.37743664490913
H	15.33800000482573	13.88976268570805	7.88155627437316
H	16.95085064171908	14.60401365273771	7.56034652917362
C	18.87723307805394	11.97582157006593	7.54359057634336
H	19.35797611724498	11.13087310419808	8.03363049173255
H	18.74751111200328	11.77815199446282	6.47887125081203
H	19.46177278676830	12.88521015114171	7.68847152536089

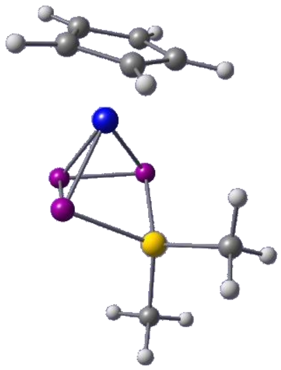

2'(Me)

ωB97X-D4/def2-TZVP (CPCM (CH_2Cl_2)): Energies/H = -3828.45093164, Free Energies/H = -3828.51554246, ZPVE/ kcal/mol = 128.91

Mo	-20.09716403890476	12.01962444501342	-6.35191106054531
As	-21.95528983559779	9.67204048197134	-7.92431029245708
P	-21.71050380955812	10.19119163037587	-5.73455408810221
P	-21.67178455456223	11.88129171308604	-8.31058190178618
P	-22.58686257571420	12.08399607875724	-6.34681640824579
H	-17.56469585035811	13.31808998479170	-5.45087298364629
C	-17.89679335312848	12.46480206496028	-6.02306166118817
C	-18.14915295610254	11.15465224325519	-5.51689732507651
H	-18.05557764936409	10.84134959212130	-4.48763770124861
H	-18.73402134337513	9.26710968533796	-6.54176539876064
C	-18.50721688152559	10.32006324016801	-6.60888051836240
C	-18.48593332672973	11.10575608493812	-7.78906503592114
H	-18.69379382982673	10.76571974680442	-8.79210395553122
C	-18.11602765778406	12.42920378432110	-7.43187710285228
H	-17.99600789792301	13.25659507530744	-8.11593048100004
C	-20.44755264125838	12.51969640678056	-4.42303845312224
O	-20.61275684437478	12.81017843794841	-3.33934208963127
C	-20.42398807464612	13.97837937327474	-6.70400841592959
O	-20.57812235202358	15.08472661347228	-6.90078638911387
C	-23.71464464589293	9.05068133218979	-8.37921987201993
H	-23.83951288681930	8.03494104694705	-8.00211773671269
H	-24.44552351644665	9.71785994528514	-7.92374485364517
H	-23.81634709580950	9.06273143481274	-9.46537100525905
C	-20.76010566216162	8.41524955880336	-8.75083727511960
H	-19.72330389443364	8.66811055571202	-8.55037701054571
H	-20.98977496543101	7.42580618097086	-8.35329801235215
H	-20.95139195924707	8.43821064559329	-9.82480204982459

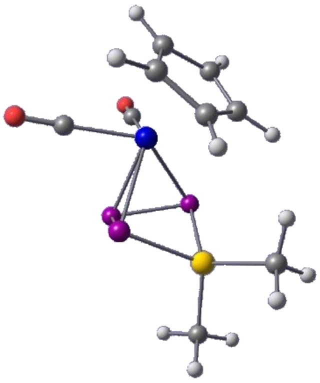

1'$_{ISO}$(Cp,Me)

ωB97X-D4/def2-TZVP (CPCM (CH_2Cl_2)): Energies/H = -5041.62613119, Free Energies/H = -5041.6825954, ZPVE/ kcal/mol = 115.02

As	21.00182695322252	12.86706229156447	13.42081815673702
Ni	20.98812127659068	10.74784057228889	12.71788446960205
P	20.39364681936602	11.59524956621810	10.69971678623506
P	19.48404609926906	13.30366796339663	11.79982213927170
P	18.86405847138592	11.17753320998011	12.03842622314640
C	21.16462407814035	8.70219910668483	12.47120110446183
H	20.53533421711664	8.11058491660884	11.82186399339727
C	22.40205524262844	9.32006998918473	12.09308862837630
C	21.94137007886587	9.83851592834397	14.29299167864512
C	20.91842297233600	8.97399850389886	13.84688284069362
C	22.87222743085564	10.04377698373918	13.20111218443317
H	23.76331161448555	10.65234703409325	13.24269441012248
H	20.06785656272577	8.63778540246576	14.42025568781311
H	22.03363878353777	10.26289922850853	15.28221258679577
H	22.85194100390653	9.28571062877234	11.11222215426443
C	20.30005628684410	13.28144565431956	15.16255540614042
H	21.01651056846721	12.94716980756185	15.91342941202485
H	20.15308839142685	14.35963119285964	15.23391721835804
H	19.35289122942535	12.75838496965162	15.28869787987951
C	22.61658198800657	13.88651998344485	13.19431236283011
H	22.98696665643604	13.72767184850664	12.18221185653773
H	22.39673157042164	14.94168050145355	13.35979849881697
H	23.34995708253877	13.53416307945322	13.92049030641644

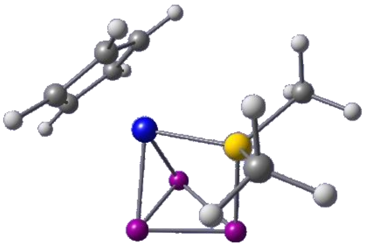

2'$_{ISO}$(Me)

ωB97X-D4/def2-TZVP (CPCM (CH_2Cl_2)): Energies/H = -3828.43904918, Free Energies/H = -3828.50333118, ZPVE/ kcal/mol = 129.04

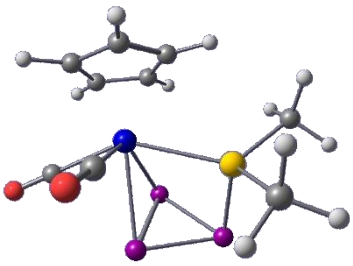

Mo	-19.20099035452533	12.35007356522331	-7.74869857470925
As	-20.81595819998431	10.88861789775451	-9.10373133353090
P	-20.91655087557539	11.59085224620482	-6.04808212350942
P	-22.45639035747795	11.22064377115328	-7.59387258209357
P	-21.59186747395604	13.21403873733179	-7.20053268008147
H	-16.46890260558810	13.53592438556411	-7.69201753828015
C	-16.95732756562397	12.57288068918983	-7.68787075630076
C	-17.35170178278960	11.82640691136944	-6.52343206086660
H	-17.22815061483773	12.13735434402195	-5.49643787355201
H	-18.26157301758379	9.79829191579991	-6.32475025681394
C	-17.88692809517728	10.58929377499491	-6.95586445857469
C	-17.81435729012635	10.56195395500599	-8.37456684473401
H	-18.10071663265279	9.73338121761753	-9.00209988437081
C	-17.24800271175172	11.77797214507532	-8.82936615541900
H	-17.05433508351312	12.03989526189950	-9.85884129544015
C	-19.03294516166254	13.97810004416177	-6.52039360374067
O	-18.93469433057314	14.88782752506872	-5.85567998032380
C	-19.43525793204642	13.69240995734702	-9.23721879625455
O	-19.51701213754388	14.44059129797244	-10.08565305256137
C	-21.43494126839619	11.45417362880649	-10.84067341640665
H	-22.25834558993482	10.80987752585200	-11.15086444089392
H	-21.77181110583859	12.48859478469423	-10.78026761702752
H	-20.61129399838709	11.37286383530766	-11.55153587793556
C	-20.61155925453694	8.97722036046204	-9.28282667170118
H	-20.26116051051546	8.56388602344727	-8.33695096913059
H	-21.58630454915221	8.55645615215013	-9.53410018616615
H	-19.90046065624816	8.75306776052340	-10.07889784058090

1

ωB97X-D4/def2-TZVP (CPCM (CH_2Cl_2)): Energies/H = -5904.33970616, Free Energies/H = -5904.44864526, ZPVE/ kcal/mol = 481.29

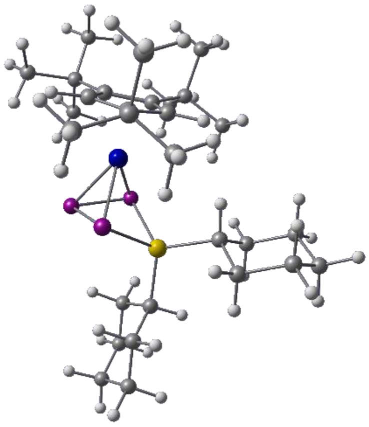

As	16.49257748186021	11.61376392723494	11.05275756505062
Ni	19.00709472627324	10.34345871269560	12.67007311043041
P	16.89673189119238	9.60224520368398	12.17223317613956
P	18.03831566456052	12.41221983479682	12.62547640677025
P	17.24283239916569	10.84475889618176	13.96242254968768
C	20.39315795663118	9.03470535426314	13.60352224975337
H	20.18957607967066	8.44857714954198	14.49252484169094
C	20.15591634018934	8.58325137396337	12.26635018653518
C	17.03420801058639	11.69161870645637	9.13343862936807
H	18.03540708221187	11.23047414663262	9.14550552500482
C	21.04413627527243	10.77047430629183	12.18909898273363
C	21.74135768944651	11.93095030441277	11.45411261366032
C	14.47162522101865	13.30884566119331	12.37190246016706
H	15.17256306924086	14.14719946694461	12.23882327492886
H	14.72904531570688	12.81953388607997	13.32672293008514
C	21.39425722937870	11.01233487029646	14.93088660917831
C	20.94414305229936	10.35911379805634	13.60972127427375
C	14.60980258018630	12.30361460254486	11.22352686568556
H	14.46379730260241	12.81933425332447	10.25895493773382
C	20.53928599813216	9.66601282450433	11.41190514089191
H	20.49910756061484	9.64028988110512	10.32877850562240
C	19.80428335940693	7.16564405148688	11.84762299038509
C	21.14808118592982	6.38710959947098	11.81750592377905
H	21.62271539062460	6.37887977246609	12.80921985853269
H	21.85064842468561	6.83485514362626	11.09974487175986
H	20.96075749377917	5.34605768803494	11.51468043160810
C	13.61844453321969	11.13848083345472	11.34668035855915
H	13.71809370872993	10.44519949424240	10.49755543240812
H	13.84238650482134	10.56330851508242	12.26154498218975
C	12.01478048965751	12.68419779833159	12.57020852892668
H	10.98823909392565	13.08221802882078	12.58190913500500
H	12.16665090478600	12.16842628017362	13.53460337825792
C	20.64962960737760	12.32832307578699	15.24418648495555
H	20.72013705040698	13.06889514127771	14.44277856889099
H	21.06756650773344	12.77550670272679	16.15874650255913
H	19.58400734507246	12.12362898419973	15.42817670004988
C	13.02501519090106	13.83216767028084	12.44645619365924
H	12.93382479055509	14.52286128078032	13.29901867720049
H	12.80637466312761	14.41831401685578	11.53697451928981
C	23.20564973638592	11.47260703480032	11.20840224983880
H	23.74663906401594	11.30280789869004	12.14833921685430
H	23.73861931210035	12.25083705005296	10.64189604206882
H	23.23163250151742	10.54204899507459	10.62339992940282
C	17.13859127897231	13.14840544541564	8.65919217424047
H	17.85500263147222	13.70664576543855	9.28151500753755
H	16.15936061058628	13.64629958435947	8.76273867728394
C	21.75138504341960	13.29363972313128	12.16790300333096
H	20.73017614736450	13.66079874584882	12.34442061552883
H	22.25914694854455	14.02189429332686	11.51847667332529
H	22.28946773432235	13.28157462305946	13.12117260694492
C	16.08700637658116	10.85480518266091	8.26076986402571
H	15.05895049294800	11.24660496753778	8.34351954745163
H	16.06558960652647	9.80946999739296	8.60539830177881
C	18.86360730086893	6.48861512265918	12.86260117005155
H	18.66629112352068	5.45410796490674	12.54608501239646
H	17.89926845405602	7.01357021739020	12.92748877529435
H	19.30600042424584	6.45190758066371	13.86797306288530
C	19.17429677113377	7.11657590959965	10.44393343703854
H	19.84369693126339	7.53955287159768	9.68132450474450
H	18.22183510689508	7.66504707908116	10.41290124341037
H	18.97367239994504	6.07104646954360	10.16876712666919
C	12.17708633050365	11.67480615517471	11.42703050063185
H	11.91643723998219	12.15745326589489	10.46914399048056

H	11.48400530005703	10.82885394770028	11.55498494609889
C	22.92574695793693	11.24200343804604	14.90864236622071
H	23.45905902926874	10.29984848804715	14.71440481953412
H	23.24658841694382	11.61852801966900	15.89121712146891
H	23.23793000757575	11.97273483701713	14.15441846540897
C	21.11379255079728	10.06570214242740	16.12102264884939
H	20.04240616504463	9.84014092481994	16.22242287406335
H	21.43875568858826	10.56227222780814	17.04631476375388
H	21.66781087481724	9.11975906705987	16.03717502435343
C	21.09115030246293	12.17154955369435	10.07200341669066
H	21.18434655479971	11.30851457770889	9.40012233326061
H	21.59617466547714	13.01787875303713	9.58524117689586
H	20.02565837624227	12.42302764273694	10.17433848351327
C	17.56913820880149	13.19586831154311	7.18105608535433
H	17.59532985851997	14.24420610970089	6.84540465923764
H	18.59818620348489	12.80714838381836	7.09147625276020
C	16.63165957345157	12.36798034098128	6.29267564372116
H	15.62653014131416	12.82517497928542	6.29801271129724
H	16.98318405651264	12.38835424638979	5.24944136643334
C	16.53007738830928	10.91946867695984	6.78701869336339
H	17.51161112050129	10.42533879008796	6.68432885622144
H	15.81957382093855	10.34807336085543	6.16983318487447

1’$_{ISO}$

ωB97X-D4/def2-TZVP (CPCM (CH_2Cl_2)): Energies/H = -5904.35267315, Free Energies/H = -5904.45497544, ZPVE/ kcal/mol = 501.34

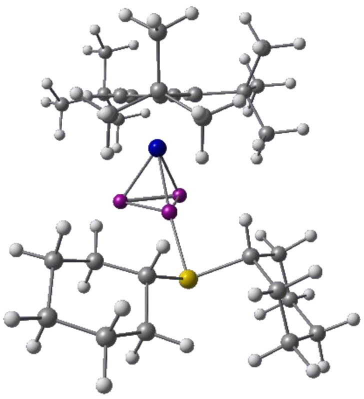

As	-21.69567807835607	15.45224141243750	-8.10832982809254
Ni	-20.04144695492498	13.93883411907382	-8.57422980212877
P	-21.73667151367026	12.56938235761628	-9.23256989635175
P	-23.02174430336252	13.63558064097571	-7.78308169496196
P	-21.20247785200517	12.52089179228358	-7.22669228690285
C	-22.59829199317595	16.38668527628481	-9.59657929200290
C	-21.62714002861249	16.18622732773858	-6.28070436097560
H	-21.34923384587542	15.25327721880061	-5.77862303435321
H	-22.23199524974135	15.78410966854826	-10.43671836691644
C	-20.52499341888978	17.20911508646808	-6.01097960637077
H	-20.34947074197477	17.19897051221583	-4.92816050274182
H	-19.58826370727756	16.89640837469323	-6.47613900193945
C	-20.89949389893237	18.62774911831913	-6.42867720273772
H	-20.10144488315146	19.31449690381619	-6.13193116357247
H	-20.97309900434091	18.68964027349291	-7.51849729026976
C	-22.98423498456932	16.64199727821960	-5.74011109807440
H	-22.91970317150807	16.58857382866108	-4.64651171619030
H	-23.77500539289859	15.94531706113451	-6.03574085855646
C	-23.34655604366028	18.07066777079172	-6.13951258073255
H	-24.27139486660491	18.36144989558554	-5.63316361817152
H	-23.55628358876482	18.11643830635555	-7.21165743615703
C	-22.22655842167414	19.05426000165046	-5.79993008888423
H	-22.49755419869875	20.05953568463825	-6.13587948559544
H	-22.10793235164723	19.10294146697342	-4.71042396811634
C	-22.20299037917597	17.84371465011830	-9.83174291343635
H	-22.55388815752844	18.46000747834428	-9.00079522575279
H	-21.12075862078462	17.95829090237877	-9.88210831004134
C	-24.11954488488661	16.23767595937815	-9.52553516063796
H	-24.50804492137249	16.78549771439479	-8.66088770565981
H	-24.40279990602249	15.19030547935714	-9.39612556621501
C	-24.76491815476592	16.77270293606465	-10.80733091953257
H	-25.85247721228949	16.68484517472509	-10.72966005324614
H	-24.45253620886233	16.14312718434996	-11.64937934961715
C	-22.84001272482001	18.35744416023341	-11.12562297729606
H	-22.55319778616123	19.40165539316613	-11.28042913184593
H	-22.44407141667000	17.78617456973572	-11.97437849580870
C	-24.36031299443903	18.21972313479251	-11.08126451316386
H	-24.75736329973977	18.86409008054702	-10.28708225329283
H	-24.80086043344761	18.56291255801634	-12.02191700872378
C	-18.46147462732128	12.72350536509389	-8.93665138860471
H	-18.55158579374379	11.64959162085800	-8.95698652605725
C	-18.20621642653271	14.81256756634115	-8.16623141611835
H	-18.03274618606176	15.65417829613175	-7.51832848781640
C	-18.45065426043109	14.91399650778923	-9.59991953877527
C	-18.22739149718170	16.27299669829327	-10.26342052496467
C	-18.12869766989422	13.47633744532380	-7.76874682378421
C	-17.65624487945046	12.97548803195046	-6.42179885420998
C	-18.76738657598762	12.98398653353846	-11.49548198156720
C	-18.48675418360788	17.41165773345577	-9.26076919786602
H	-19.46160280021338	17.32369435656959	-8.78406973015251
H	-18.45473219682150	18.36635060933315	-9.78921249163787
H	-17.72886650817609	17.44898724098614	-8.47700456475565
C	-18.34014061440584	13.73645961467033	-5.27978046569409
H	-18.18884690910270	14.81478746826288	-5.37203759210907
H	-17.91900151225485	13.41800237833534	-4.32268035741330
H	-19.41498897167930	13.54434172380068	-5.26240837730979
C	-18.59547185010596	13.57763942916619	-10.09116381696914
C	-19.06590238198682	16.54996978499923	-11.51194982228729
H	-18.81078564717408	15.89665111795143	-12.34241334798824
H	-18.87973778782398	17.57617565428752	-11.83769012903467
H	-20.13252670313635	16.45077634347760	-11.30501114674448
C	-20.06005989247521	13.41649568555381	-12.20659392069464
H	-20.92131367202987	12.89453025784056	-11.78897527408516

H	-19.99262538950019	13.14721688983655	-13.26450808953541
H	-20.25197903159482	14.48180308065653	-12.13842711523647
C	-17.54386214574037	13.34575151088194	-12.35627267762945
H	-17.53230005309311	14.39353504705605	-12.64910789125934
H	-17.56486664602401	12.75009598912195	-13.27248255916178
H	-16.61365704240577	13.12362402371414	-11.82647689364684
C	-16.72664167165906	16.34099234124838	-10.61873781758161
H	-16.11402141617693	16.18425108854660	-9.72689221255412
H	-16.49921569074019	17.33352485768629	-11.01758213619949
H	-16.44305550619791	15.59938973319746	-11.36313582482725
C	-17.87960564453265	11.46908351993668	-6.26833442373654
H	-18.93690344209050	11.20267146887779	-6.31885661286171
H	-17.49787274697542	11.14434432694054	-5.29731486916660
H	-17.34553561115722	10.91039448990964	-7.04132126178902
C	-16.14052374953636	13.25154562228118	-6.37316375558815
H	-15.62672202374157	12.74407826642583	-7.19429429018530
H	-15.72849264631582	12.88352505638098	-5.42976929362174
H	-15.93415217695722	14.32250722701159	-6.44637649880906
C	-18.80539344863967	11.44852639433199	-11.41401293091715
H	-17.87175805894102	11.03822526871573	-11.02076983695855
H	-18.94796920018319	11.05212236230654	-12.42186405238763
H	-19.63389910391126	11.09008978854528	-10.79819004463873

References

[1] https://omics.pnl.gov/software/molecular-weight-calculator (07.04.2025).

[2] E. Mädl, G. Balázs, E. V. Peresypkina, M. Scheer, “Unexpected Reactivity of (η^5-1,2,4-$^tBu_3C_5H_2$)Ni(η^3-P_3) towards Main Group Nucleophiles and by Reduction”, *Angew. Chem. Int. Ed.* **2016**, *55*, 7702.

[3] O. J. Scherer, H. Sitzmann, G. Wolmershäuser, “Umsetzung von P_4 mit (η^5-C_5H_5)$(CO)_2$Mo≡Mo$(CO)_2$(η^5-C_5H_5) zu den tetraedrischen molybdänkomplexen Pn[Mo$(CO)_2$(η^5-C_5H_5)]$_{4-n}$ (n = 2,3)”, *J. Organomet. Chem.* **1984**, *268*, C9.

[4] M. Gorzellik, H. Bock, L. Gang, B. Nuber, M. L. Ziegler, “Darstellung und charakterisierung von zweikernigen oxo-komplexen des molybdän wolfram mit chalkogenen (O, S, Se, Te) als brückenliganden”, *J. Organomet. Chem.* **1991**, *412*, 95.

[5] M. Gonsior, I. Krossing, N. Mitzel, “A Thallium Coated Dianion: Trigonal Bipyramidal $[F_2Al(OR)_3]^{2-}$ Coordinated to Three Tl^+ Cations in the Ion Pair $[Tl_3F_2Al(OR)_3]$+$[Al(OR)_4]$— [R = $CH(CF_3)_2$]”, *Z. anorg. allg. Chem.* **2002**, *628*, 1821.

[6] R. P. Hughes, D. C. Lindner, A. L. Rheingold, G. P. A. Yap, “Synthesis and Structure of the Thallium(I) Salt of the Tetrakis{3,5-bis(trifluoromethyl)phenyl}borate Anion”, *Inorg. Chem.* **1997**, *36*, 1726.

[7] W. Steinkopf, H. Dudek, S. Schmidt, *Ber. dtsch. Chem. Ges. A/B* **1928**, *61*, 1911.

[8] C. Riesinger, L. Dütsch, G. Balázs, M. Bodensteiner, M. Scheer, “Cationic Functionalisation by Phosphenium Ion Insertion”, *Chem. Eur. J.* **2020**, *26*, 17165.

[9] I. Bernal, H. Brunner, W. Meier, H. Pfisterer, J. Wachter, M. L. Ziegler, “Ligand Extrusion from the As_4S_4 -Cage by $[C_5Me_5(CO)_2Mo]_2$: Formation of $(C_5Me_5)_2Mo_2(CO)_4(\mu,\eta^2\text{-}As_2)$, $C_5Me_5(CO)_2Mo(\eta^3\text{-}As_3)$, and $(C_5Me_5)_2Mo_2As_2S_3$”, *Angew. Chem. Int. Ed. Engl.* **1984**, *23*, 438.

[10] Agilent, CrysAlisPro **2014**, Agilent Technologies Ltd, Yarnton, Oxfordshire, England”.

[11] O. V. Dolomanov, L. J. Bourhis, R. J. Gildea, J. A. K. Howard, H. Puschmann, “OLEX2 : a complete structure solution, refinement and analysis program”, *J Appl Crystallogr* **2009**, *42*, 339.

[12] G. M. Sheldrick, “SHELXT - integrated space-group and crystal-structure determination”, *Acta Cryst. A* **2015**, *71*, 3.

[13] G. M. Sheldrick, “Crystal structure refinement with SHELXL”, *Acta Cryst. C* **2015**, *71*, 3.

[14] F. L. Hirshfeld, “Bonded-atom fragments for describing molecular charge densities”, *Theoret. Chim. Acta* **1977**, *44*, 129.

[15] S. C. Capelli, H.-B. Bürgi, B. Dittrich, S. Grabowsky, D. Jayatilaka, “Hirshfeld atom refinement”, *IUCrJ* **2014**, *1*, 361.

[16] F. Meurer, F. Kleemiss, C. Riesinger, G. Balázs, V. Vuković, I. G. Shenderovich, C. Jelsch, M. Bodensteiner, “Probing the Isolobal Relation between Cp’’’NiP_3 and White Phosphorus by Experimental Charge Density Analysis”, *Chem. Eur. J.* **2024**, *30*, e202303762.

[17] F. Kleemiss, O. V. Dolomanov, M. Bodensteiner, N. Peyerimhoff, L. Midgley, L. J. Bourhis, A. Genoni, L. A. Malaspina, D. Jayatilaka, J. L. Spencer et al., “Accurate crystal structures and chemical properties from NoSpherA2”, *Chem. Sci.* **2020**, *12*, 1675.

[18] F. Neese, “The ORCA program system”, *WIREs Comput Mol Sci* **2012**, *2*, 73.

[19] F. Neese, “Software update: the ORCA program system, version 4.0”, *WIREs Comput. Mol. Sci.* **2018**, *8*.

[20] F. Neese, F. Wennmohs, U. Becker, C. Riplinger, “The ORCA quantum chemistry program package”, *J. Chem. Phys.* **2020**, *152*, 224108.

[21] F. Neese, “Software update: The ORCA program system—Version 5.0”, *WIREs Comput. Mol. Sci.* **2022**, *12*.

[22] F. Neese, “The SHARK integral generation and digestion system”, *J. Comput. Chem.* **2023**, *44*, 381.
[23] J.-D. Chai, M. Head-Gordon, “Long-range corrected hybrid density functionals with damped atom-atom dispersion corrections”, *Phys. Chem. Chem. Phys.* **2008**, *10*, 6615.
[24] J.-D. Chai, M. Head-Gordon, “Systematic optimization of long-range corrected hybrid density functionals”, *J. Chem. Phys.* **2008**, *128*, 84106.
[25] F. Weigend, R. Ahlrichs, “Balanced basis sets of split valence, triple zeta valence and quadruple zeta valence quality for H to Rn: Design and assessment of accuracy”, *Phys. Chem. Chem. Phys.* **2005**, *7*, 3297.
[26] M. Garcia-Ratés, F. Neese, “Effect of the Solute Cavity on the Solvation Energy and its Derivatives within the Framework of the Gaussian Charge Scheme”, *J. Comput. Chem.* **2020**, *41*, 922.
[27] F. de Proft, R. Vivas-Reyes, A. Peeters, C. van Alsenoy, P. Geerlings, “Hirshfeld partitioning of the electron density: atomic dipoles and their relation with functional group properties”, *J. Comput. Chem.* **2003**, *24*, 463.
[28] S. Grabowsky, “Complementary Bonding Analysis”, Walter de Gruyter, Bern, **2021**.
[29] R. F. W. Bader, “A quantum theory of molecular structure and its applications”, *Chem. Rev.* **1991**, *91*, 893.
[30] W. F. Kuhs, “The Anharmonic Temperature Factor in Crystallographic Structure Analysis”, *Aust. J. Phys.* **1988**, *41*, 369.
[31] T. Lu, F. Chen, “Multiwfn: a multifunctional wavefunction analyzer”, *J. Comput. Chem.* **2012**, *33*, 580.
[32] S. Grimme, A. Hansen, S. Ehlert, J.-M. Mewes, “r2SCAN-3c: A “Swiss army knife” composite electronic-structure method”, *J. Chem. Phys.* **2021**, *154*, 64103.
[33] “Chemcraft – graphical software for visualization of quantum chemistry computations.” https://www.chemcraftprog.com” (**2024**).
[34] Y.-S. Lin, G.-D. Li, S.-P. Mao, J.-D. Chai, “Long-Range Corrected Hybrid Density Functionals with Improved Dispersion Corrections”, *J. Chem. Theory Comput.* **2013**, *9*, 263.
[35] E. Caldeweyher, S. Ehlert, A. Hansen, H. Neugebauer, S. Spicher, C. Bannwarth, S. Grimme, “A generally applicable atomic-charge dependent London dispersion correction”, *J. Chem. Phys.* **2019**, *150*, 154122.
[36] J. Tomasi, B. Mennucci, R. Cammi, “Quantum mechanical continuum solvation models”, *Chem. Rev.* **2005**, *105*, 2999.
[37] V. Ásgeirsson, B. O. Birgisson, R. Bjornsson, U. Becker, F. Neese, C. Riplinger, H. Jónsson, “Nudged Elastic Band Method for Molecular Reactions Using Energy-Weighted Springs Combined with Eigenvector Following”, *J. Chem. Theory Comput.* **2021**, *17*, 4929.
